# Two Distinct Coagulase-Dependent Barriers Protect *Staphylococcus aureus* from Neutrophils in a Three Dimensional *in vitro* Infection Model

**DOI:** 10.1371/journal.ppat.1002434

**Published:** 2012-01-12

**Authors:** Christoph Guggenberger, Christiane Wolz, Julie A. Morrissey, Jürgen Heesemann

**Affiliations:** 1 Max von Pettenkofer-Institut, Ludwig-Maximilians-University of Munich (LMU), Munich, Germany; 2 Interfaculty Institute of Microbiology and Infection Medicine, University of Tübingen, Tübingen, Germany; 3 Department of Genetics, University of Leicester, Leicester, United Kingdom; University of California, San Francisco, United States of America

## Abstract

*Staphylococcus* aureus is a pyogenic abscess-forming facultative pathogenic microorganism expressing a large set of virulence-associated factors. Among these, secreted proteins with binding capacity to plasma proteins (e.g. fibrinogen binding proteins Eap and Emp) and prothrombin activators such as Coagulase (Coa) and vWbp are involved in abscess formation. By using a three-dimensional collagen gel (3D-CoG) supplemented with fibrinogen (Fib) we studied the growth behavior of *S. aureus* strain Newman and a set of mutants as well as their interaction with mouse neutrophils by real-time confocal microscopy. In 3D-CoG/Fib, *S. aureus* forms microcolonies which are surrounded by an inner pseudocapsule and an extended outer dense microcolony-associated meshwork (MAM) containing fibrin. Coa is involved in formation of the pseudocapsule whereas MAM formation depends on vWbp. Moreover, *agr*-dependent dispersal of late stage microcolonies could be observed. Furthermore, we demonstrate that the pseudocapsule and the MAM act as mechanical barriers against neutrophils attracted to the microcolony. The thrombin inhibitor argatroban is able to prevent formation of both pseudocapsule and MAM and supports access of neutrophils to staphylococci. Taken together, this model can simulate specific stages of *S. aureus* abscess formation by temporal dissection of bacterial growth and recruitment of immune cells. It can complement established animal infection models in the development of new treatment options.

## Author Summary

*Staphylococcus aureus* is one of the most frequent pathogens causing divers localized and metastatic abscess-forming infections. Here we studied the role of the staphylocoagulases Coa and vWbp in the formation of microcolony-associated fibrin structures. By using a three-dimensional collagen gel (3D-CoG) supplemented with human fibrinogen as a growth environment for staphylococci and as a neutrophil migration matrix, we were able to demonstrate that Coa is involved in producing a fibrin-containing pseudocapsule wrapping the staphylococcal microcolony whereas vWbp is required for establishing an extended outer fibrin meshwork. The pseudocapsule and the outer meshwork hinder neutrophils from attacking the staphylococci. Addition of the thrombin inhibitor argatroban prevents conversion of fibrinogen to fibrin and thus abolishes barrier formation. This *in vitro* model provides us with new options to study formation as well as prevention of staphylococcal abscesses under tissue-like conditions.

## Introduction

*Staphylococcus aureus* is a common human colonizer of skin and nasopharynx. Under conditions of impaired immune defense *S. aureus* carriers are at increased risk to develop severe infections ranging from localized soft tissue to invasive infections such as endocarditis, metastatic infections of joints, kidneys and lungs with progression to sepsis [1]. Treatment of staphylococcal infections has been further complicated by the massive development of antibiotic resistances in recent years [2]. Adherence to host epithelium is critical to colonization in the carrier stage as well as to invasion and metastatic dissemination. In regard of this complex host-pathogen interaction *S. aureus* has evolved a highly adaptive and versatile strategy to survive and replicate in beneficial as well as in hostile environments. *S. aureus* is equipped with a large set of fine-tuned virulence-associated genes of which gene products can be roughly classified into several groups, among those are adhesins/invasins (which are mainly involved in the interaction with extracellular matrix (ECM) proteins), pore-forming toxins, superantigens and immune evasion factors [3]. The adhesin/invasin comprises a subgroup of cell wall anchored proteins, termed MSCRAMMs (Microbial Surface Components Recognizing Adhesive Matrix Molecules) and a subgroup of SERAMs (Secretable Expanded Repertoire Adhesive Molecules) which are released but mainly surface-associated proteins [4], [5]. The MSCRAMM subgroup includes fibronectin binding proteins (FnbpA, FnbpB), fibrinogen/fibrin binding proteins such as the clumping factor A and B (ClfA, ClfB), the collagen binding protein (Cna) and *Staphylococcus* protein A (Spa), which binds immunoglobulin G (IgG) and von Willebrand factor (vWF) [4], [6]. The SERAM subgroup also includes fibrinogen/fibronectin binding proteins such as the extracellular adherence protein (Eap) and the extracellular matrix binding protein (Emp) [5], [7], [8] but also prothrombin-activating proteins such as coagulase (Coa) and von Willebrand factor binding protein (vWbp) [9], [10]. The latter are able to activate prothrombin in a non-proteolytic manner, opposed to physiological prothrombin activation. The resulting Coa- or vWbp-prothrombin complex converts soluble fibrinogen into insoluble fibrin fibers [9], [11].

At a first glance MSCRAMMs and SERAMs may be of redundant function in the context of colonization and infection. On the other hand there must be a selective pressure for maintenance of virulence-associated genes with apparent redundant functions, suggesting different roles in the complex life style of *S. aureus*.

The virulon of *S. aureus* is orchestrated by different global regulatory systems such as Agr, Sar and Sae, all of which sense environmental changes [12]. The Sae regulatory system (*S. aureus* exoprotein expression) seems not to affect the Agr and Sar systems and controls the expression of genes encoding hemolysins (*hla* and *hlb*) and several MSCRAMMs and SERAMs [13].

To investigate the contribution of these virulence-associated factors to disease initiation and progression, various *in vitro* and *in vivo* infection models have been established. Recently, the molecular mechanisms of *S. aureus* Newman abscess formation in the mouse infection model could be elucidated by using defined mutants deficient in production of e.g. Coa, vWbp, Eap and Emp [14], [15]. It could be demonstrated that mature abscesses in the kidney are composed of a core structure of the staphylococcal abscess community (SAC) which is enclosed by a pseudocapsule of fibrin deposits and a layer of neutrophils in the periphery. This supports a novel concept in abscess formation by pointing to an exploitation of host clotting machinery by staphylococcal virulence factors in order to establish a protective niche for the pathogen [16].

However, it is difficult to assess the role of single MSCRAMM or SERAM proteins during infection *in vivo* because of the complexity of the infection process and coevolution-driven host specificity of *S. aureus* (e.g. species specificity of coagulase and human-specific hemoglobin utilization [17], [18]).

In this study, we focused on growth behavior of *S. aureus* strain Newman in a three dimensional collagen gel setup (3D-CoG) and the interaction with polymorphonuclear leukocytes (PMNs or neutrophils). The 3D-CoG is a suitable matrix to study neutrophil migration and microbial growth under tissue-like conditions [19]–[21]. The 3D-CoG can be modified by adding purified ECM or plasma proteins of interest in order to study the dynamics of bacterial growth and interaction with these proteins as well as their role in supporting or inhibiting bacteria-neutrophil interaction. We used this tissue-like system to study the formation of a fibrin capsule/meshwork surrounding *S. aureus* clusters by using defined mutants of strain Newman but also other clinical isolates. In a second step we analyzed the implications of these barrier-like structures for the infiltration of neutrophils and for the subsequent degradation of bacterial microcolonies.

## Results

### *S. aureus* Microcolonies Are Surrounded by Two Concentric Fibrin Structures

**Figure 1:**
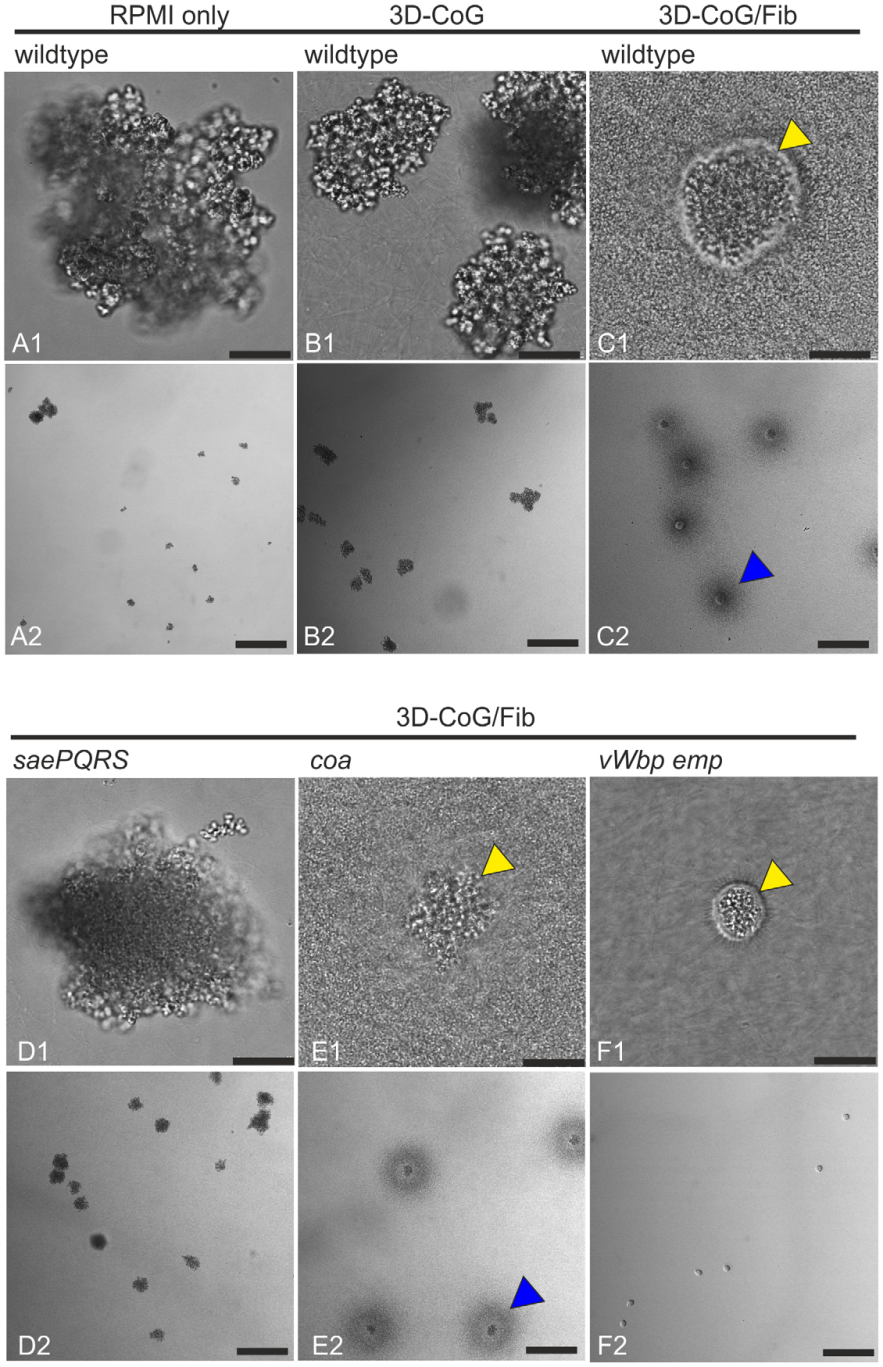
Growth phenotypes of *S. aureus* Newman strains in different environments. Growth phenotypes of *S. aureus* Newman strains under different growth conditions were analyzed 16 h after inoculation without agitation. Growth in RPMI 1640 leads to cluster formation of variable size (A). Growth in 3D-CoG also leads to cluster formation (B). Addition of 3 mg/ml fibrinogen to the medium (3D-CoG/Fib) resulted in the formation of discrete microcolonies of uniform diameter, surrounded by an inner pseudocapsule (yellow arrowheads) and an outer dense microcolony-associated meshwork MAM (blue arrowheads) (C). The *sae* mutant (Newman-29) formed clusters comparable to wildtype, even in 3D-CoG/Fib (D). A *coa* mutant formed microcolonies which were irregularly shaped in comparison to the wildtype, MAM formation was unaffected (E). A *vWbp emp* double mutant was unaffected in formation of the inner pseudocapsule but was devoid of any outer MAM (F). A1-F1: 40x oil immersion objective, scale bar 25 µm. A2-F2: 10x objective, scale bar 200 µm.

In a first approach to establish a collagen gel-system (3D-CoG) for studying staphylococci-neutrophil interaction we analyzed the growth behavior of strain Newman. When growing *S. aureus* Newman for 16 h in RPMI 1640 medium without agitation, single bacterial cells gave rise to irregularly shaped bacterial clusters of variable size (Fig. 1A). In the next step we mixed monodispersed staphylococci with neutralized collagen type I solutions, followed by incubation at 37°C. Gelling occurred within 45 min and resulted in a rigid matrix which was then overlaid with medium. Staphylococci replicated and formed clusters similar to those observed in RPMI 1640 without collagen after 16 h (Fig. 1B). The 3D-CoG meshwork was not degraded by staphylococci even after days (data not shown). Moreover, we could not observe specific interaction of staphylococci with collagen fibers by confocal microscopy. Thus, a 3D-CoG system is suitable for studying growth of *S. aureus* in a rigid matrix by microscopy.

**Figure 2:**
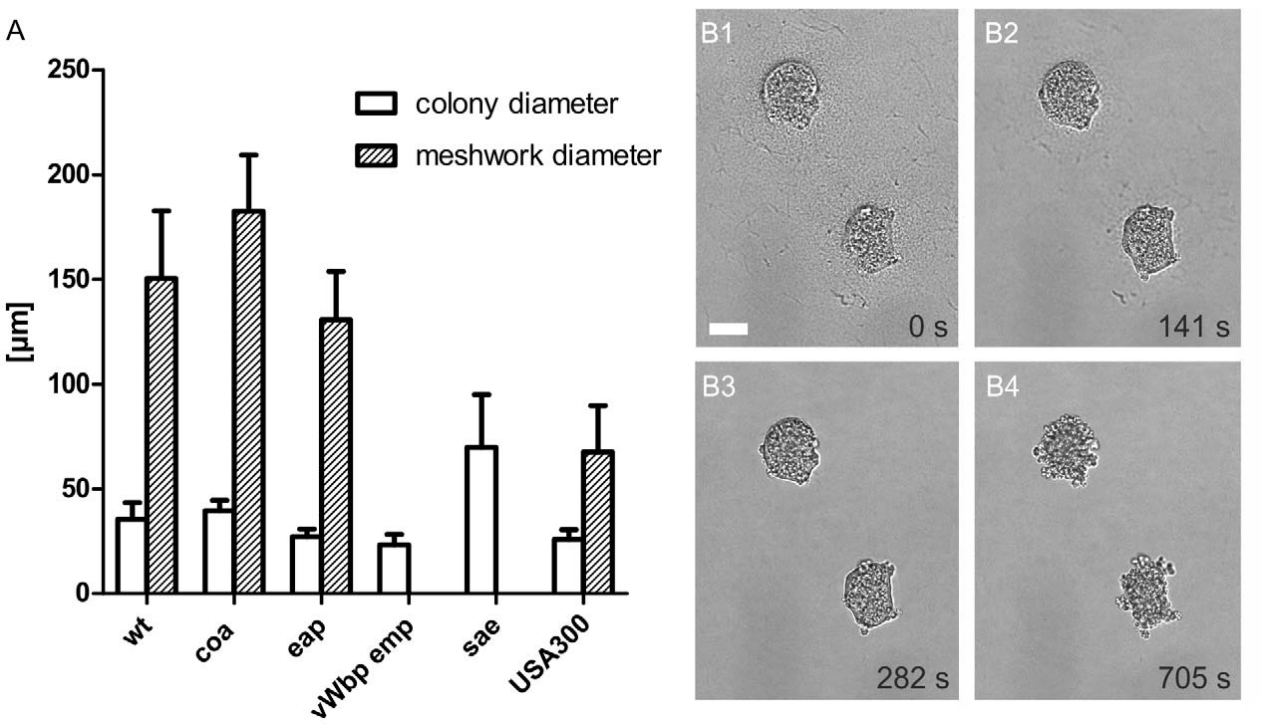
Microcolony and MAM diameter of *S. aureus* Newman strains after growth in 3D-CoG/Fib. The average diameter of microcolonies and MAM was determined after 16 h of growth in 3D-CoG/Fib (A). Wildtype (wt), *coa* mutant and *eap* mutant formed microcolonies and MAM of comparable size. The *vWbp emp* double mutant did not form any MAM, despite being unaffected in pseudocapsule formation. A *sae* mutant (Newman-29) neither formed pseudocapsules nor MAM but grew in clusters significantly larger than wt colonies. USA300 microcolonies were comparable in size to wt, pseudocapsule formation was present, but the diameter of the MAM was significantly smaller compared to wt. Data are averaged from at least three independent experiments. Addition of plasmin (8 µg/ml) led to rapid degradation of both pseudocapsule and MAM surrounding *S. aureus* Newman colonies grown in 3D-CoG/Fib for 17h (B1–B4). The time stamp in the single panels is relative to plasmin addition, scale bar 25 µm.

In order to supply a more tissue-like environment we added fibrinogen corresponding to normal serum concentration (3 mg/ml) to the growth medium (3D-CoG/Fib). This led to dramatic changes in the growth behavior of staphylococci (Fig. 1C):

Firstly, single staphylococci gave rise to discrete microcolonies of uniform size after 16 h of growth which were surrounded by a spherical pseudocapsule (about 35 µm in diameter and 1–3 µm in thickness, Fig. 2A). The encapsulated microcolonies consisted of densely packed staphylococci and appeared to be free of collagen fibers.

Secondly, the pseudocapsules were embedded into an outer dense microcolony-associated meshwork (MAM) surrounding the microcolonies (approximately 150 µm in diameter, Fig. 2A). Both of these concentric structures, the inner pseudocapsule and the outer MAM, were only formed in the presence of fibrinogen. The observation that these structures - in contrast to collagen fibers - were rapidly degraded after the addition of plasmin (8 µg/ml) suggests that they are at least in part composed of fibrin (Fig. 2B and Video S1).

As the observed pseudocapsule and the MAM appeared to consist of fibrinogen/fibrin components, we assumed an involvement of secreted proteins belonging to the SERAM family [5]. Most, if not all of these genes have been shown to be transcriptionally activated by the *saeRS* two-component system [13], [22]. Therefore, the Newman *sae* mutant with severe repression of SERAM-encoding genes (Newman-29, [13]) was analyzed for its growth behavior in 3D-CoG/Fib (Fig. 1D and 2A). Both pseudocapsule and MAM formation were completely abrogated; the growth phenotype resembled cluster formation of strain Newman in 3D-CoG without fibrinogen. Of note, cell wall- anchored fibrinogen binding proteins ClfA and ClfB are not affected in the *sae* mutant [23]. Thus, we reasoned that SERAM family members activated by the *saeRS* two-component system could be involved in the formation of these putatively fibrin-based structures.

**Figure 3:**
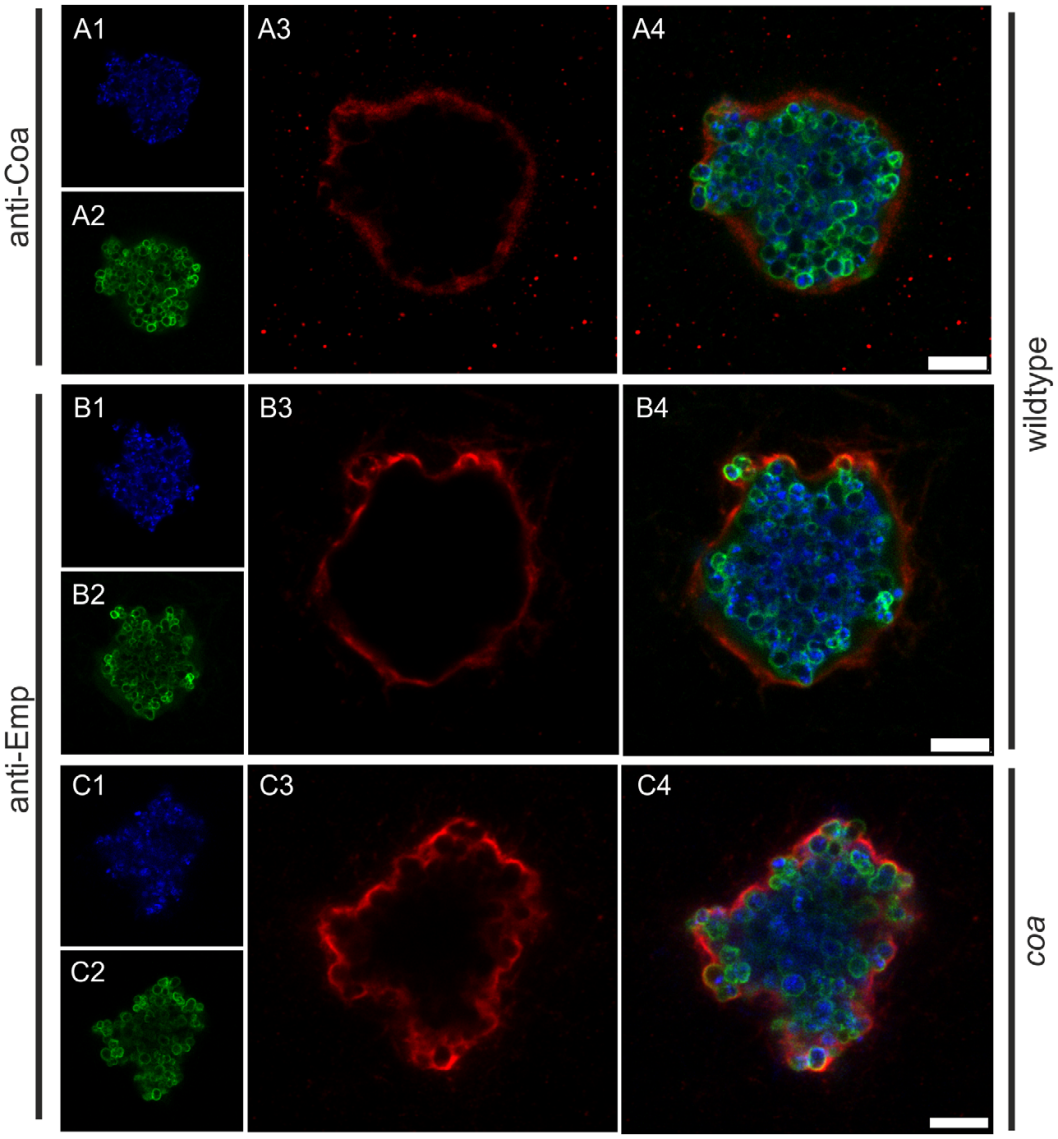
Localization of Coa and Emp to *S. aureus* pseudocapsules. Coa and Emp were detected in pseudocapsules by immunocytochemistry using rabbit antibodies specific for coagulase (anti-Coa, A) or Emp (anti-Emp, B and C). The pseudocapsule of the *coa* mutant is more irregularly shaped than its wildtype counterpart, illustrated by anti-Emp staining (C). Staphylococci were grown in 3D-CoG/Fib for 16 h and then fixed with 4% paraformaldehyde. Primary antibodies were detected by Alexa Fluor 555 anti-rabbit antibodies. Staphylococci were detected by staining DNA with DAPI (A1, B1, C1) and N-acetyl-glucosamine with FITC-Lectin (*T. vulgaris*) (A2, B2, B3). Staining of Coa and Emp was specific as the *coa* and *vwbp emp* mutant were not stained with the respective antisera (data not shown). Scale bar 7.5 µm. The images are representative of three independent experiments.

Immunostaining of Emp and Coa revealed their localization on or within the pseudocapsule (Fig. 3). In order to elucidate the pseudocapsule and MAM formation process, we analyzed a set of Newman mutants in *coa*, *vWbp*, *eap*, and *emp*. Emp and Eap production were confirmed by SDS surface extracts (Fig. S1), Coa and vWbp were detected in culture supernatants and confirmed by MALDI-TOF (Fig. S1). A *coa* mutant was still able to form pseudocapsules and MAM, although the pseudocapsules and the enclosed microcolonies were considerably more irregularly shaped than those of the parental Newman strain (Fig. 1E, 2A and 3C). Obviously, Coa was partially involved in the formation of the pseudocapsule.

The *vWbp emp* double mutant was able to form a pseudocapsule similar to that of the parent strain (Fig. 1F and 2A). However, the microcolonies were completely devoid of the MAM. Ectopic complementation studies with a set of plasmids (encoding *vWbp* or *emp* or both) showed that this phenotype was solely dependent on vWbp (Fig. S2). The increased MAM diameter compared to the parental strain could be explained by the increased secretion of vWbp due to the multicopy ectopic complementation (compare Fig. S1).

Interestingly, the *eap* mutant and an *ica* mutant were phenotypically indiscernible from the parent strain when grown in suspension or in 3D-CoG/Fib (Fig. 2A and data not shown).

Taken together, *S. aureus* Newman microcolonies grown in a 3D-CoG matrix in the presence of fibrinogen were surrounded by two distinguishable concentric structures: an inner pseudocapsule and an outer dense microcolony-associated meshwork which we termed MAM.

### Presence of Pseudocapsules and MAMs in Clinical Isolates

**Figure 4:**
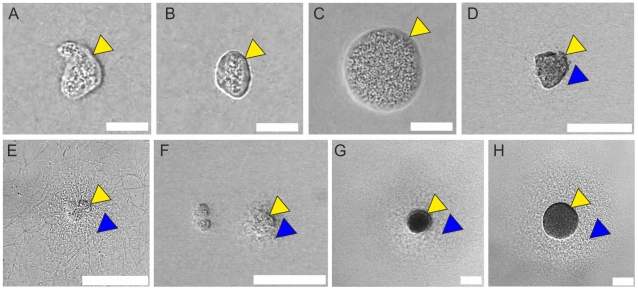
Presence of pseudocapsule and MAM structures in various clinical isolates. Clinical *S. aureus* isolates were cultivated as described for strain Newman and evaluated after 16 h of growth. MSSA strain MP9-11 (A). MSSA strain MP3-11 (B). MSSA strain MP6-11 (C). MRSA strain MP10-11 (D). CA-MRSA strain USA300 (E). MSSA strain MP2-11 (F). MRSA strain ST239-CC8 (G). MSSA strain MP1-11 (H). Pseudocapsules (yellow arrowheads) and MAM-like structures (blue arrowheads) are indicated. A–C: scale bar 20 µm. D–H: scale bar 75 µm.

It has been reported that strain Newman is dysregulated in SERAM-production because of an amino acid exchange in SaeS, the sensor kinase of the *saeRS* two-component system, compared to other *S. aureus* strains [13]. Therefore, we also assessed several clinical *S. aureus* isolates for their growth behavior (Fig. 4), among these MSSA strains freshly isolated from several patients (blood, sputum, abscesses) and two MRSA reference strains (USA300 FPR3757 [24] and ST239-CC8 [25]). Strikingly, pseudocapsule formation was found in all of these strains, although there was a broad spectrum of microcolony size variations. Furthermore, the pseudocapsules of some strains were less regularly shaped in comparison to strain Newman. We also found MAM-like structures in 5 of 11 isolates, the size and regularity of which were also strain-dependent (e. g. for USA300 compare Fig. 2A and 4E). Some strains forming much larger microcolonies than Newman caused complete solidification of the medium supernatant within the first 16 h which was observed with strain Newman only after about 20–40h.

### Dispersal of Microcolonies as a Result of Fibrin Degradation

**Figure 5:**
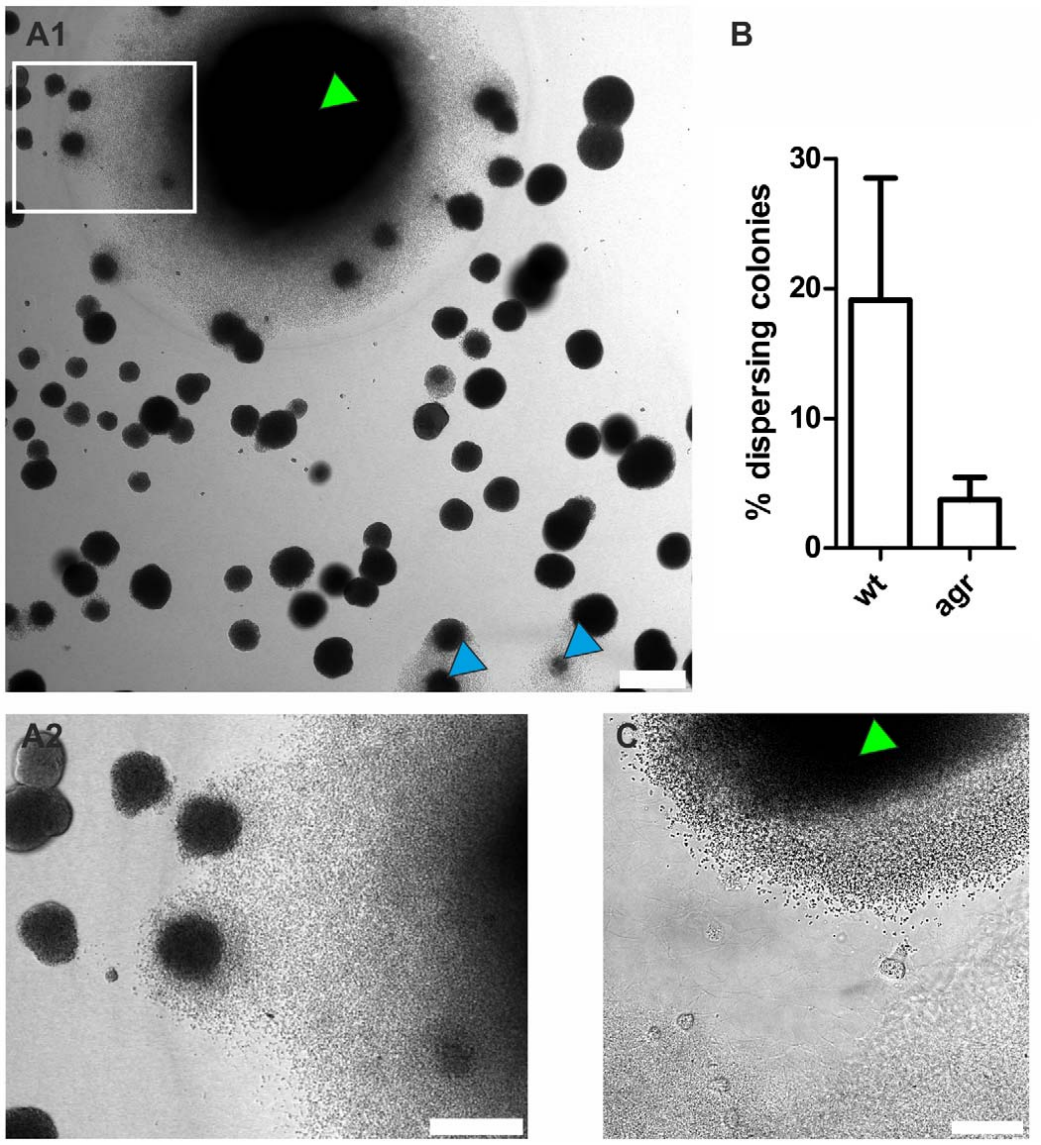
Degradation of fibrin-structures in later growth phases. At later time points (here: 43 h after inoculation) single *S. aureus* Newman microcolonies (green arrowhead) started massive growth and dispersal by degradation of fibrin structures (A). Also comparatively small microcolonies are able to switch to this mode (blue arrowhead). A2 is a magnification of the inset in A1. An *agr* mutant is significantly less prone to degrade fibrin structures compared to the wildtype (B). The percentage of dispersing colonies was evaluated after 3 days by counting the respective microcolonies in a set of n independent wells (containing between 60 and 120 microcolonies per counted area, wt n = 11, agr n = 12, *p<0.0001*). Similar observations were made with CA-MRSA strain USA300 (C). A time lapse video of the lower left area in C is shown in Video S2. Scale bar sizes: A 150 µm, B 75 µm, C 50 µm.

We could show that at later time points (>20–40 h) single *S. aureus* Newman microcolonies transited from relative growth arrest to massive growth and dispersal (Fig. 5). This event was accompanied by degradation of both the pseudocapsule and the MAM, probably by releasing a soluble fibrin-specific protease as the underlying collagen matrix was not degraded. This factor also degraded the fibrin structures of nearby colonies without inducing growth immediately (data not shown and compare Video S2). Similar to strain Newman, fibrin degradation was observed with USA300 microcolonies (Fig. 5C and Video S2).

Comparison of Newman wildtype with the *agr* mutant revealed the involvement of the *agr* system in microcolony dispersal (Fig. 5B). Moreover, addition of the fibrinolysis inhibitors aprotinin or tranexamic acid [26] to the growth medium suppressed this behavior completely, even up to 6d after inoculation, while formation of pseudocapsule and MAM was unaffected (Fig. S3).

### Bacterial Microcolonies Are Protected from Host Immune Cells by the *vWbp*-dependent MAM

**Figure 6:**
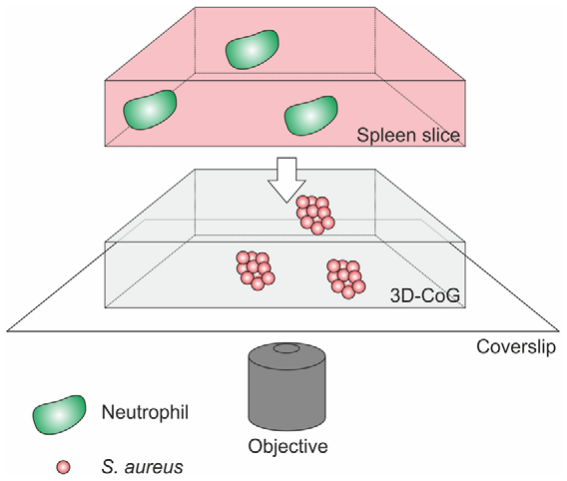
Schematical drawing of the setup. Staphylococci are grown in 3D-CoG/Fib in wells for 16 h in the absence of neutrophils. Then the medium is removed, stainings (e. g. Sytox Blue) can be applied and the 3D-CoG is overlaid with a 300 µm thin native spleen slice. Neutrophils migrate from the tissue slice into the 3D-CoG where they interact with staphylococci. The bottom of the well is suitable for confocal microscopy.

Bacterial pathogens shield themselves from humoral and cellular factors of the host defense system by formation of exopolysaccharide or proteinaceous capsules. Therefore, we assessed if the *S. aureus* pseudocapsule and the MAM also affect the interaction of neutrophils with staphylococcal microcolonies. For this purpose we established a novel method for applying neutrophils to our 3D-CoG system. *Lys-EGFP* mice [27] were used, in which mature neutrophils express eGFP under the control of the lysM promoter. Native spleen explants of *lys-EGFP* mice were cut into thin slices (300 µm) using a vibrating blade microtome. These slices were then layered upon the surface of the 3D-CoG and fluorescent neutrophils presumably originating from the red pulp migrated into the collagen matrix (see also Fig. 6). This technique of direct transfer of neutrophils from splenic tissue to 3D-CoG avoids cell manipulation required for neutrophil isolation from blood or bone marrow which may affect neutrophil behavior. By this approach we could show that almost exclusively eGFP-positive cells migrated into the 3D-CoG. These cells were identified as neutrophils by immunostaining of the specific marker Ly-6G (Fig. S4). Non-fluorescent cells inside the 3D-CoG were only rarely observed.

In order to analyze the effects of pseudocapsule and MAM on neutrophils, staphylococci were pre-grown in 3D-CoG/Fib for 16–17 h and then challenged with murine neutrophils.

**Figure 7:**
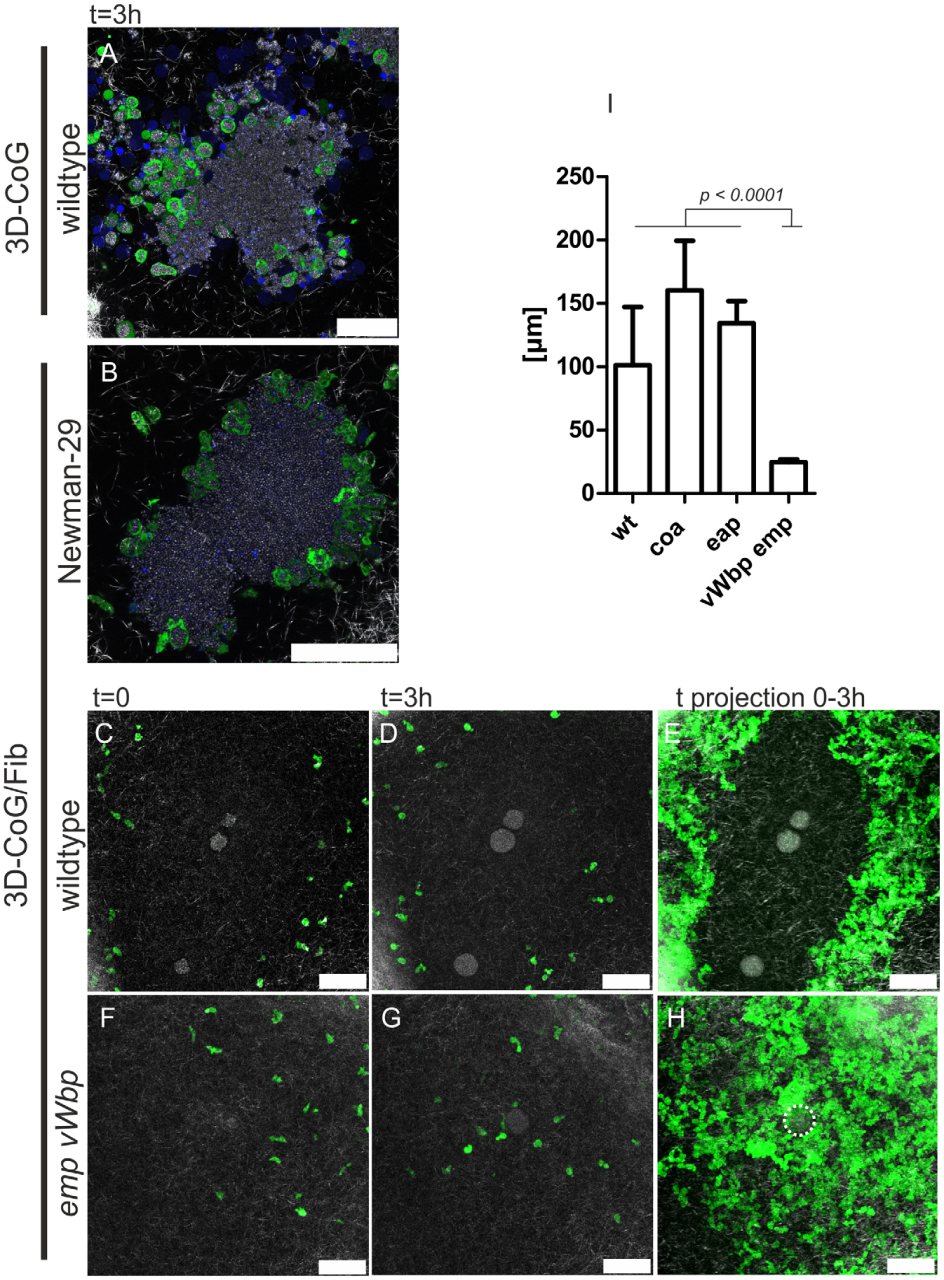
*S. aureus* Newman microcolonies are protected from neutrophils by MAM. Neutrophils approached Newman wildtype microcolonies grown in 3D-CoG and immediately started phagocytosis (A, picture 3 h after challenge with neutrophils). The *sae* mutant (Newman-29) grown in 3D-CoG/Fib was attacked in the same way (B, picture 3 h after challenge with neutrophils). Wildtype microcolonies grown in 3D-CoG/Fib were not approached by neutrophils within 3 h (C, D). This can be depicted more clearly by time projection (0–3 h) of single frames from Video S3 (E). In contrast to this, the *vWbp emp* double mutant was readily approached by neutrophils which were only held back by the pseudocapsule (F–H, see Video S5). This neutrophil-free halo surrounding the microcolonies was measured and revealed a correlation with the MAM (I). Green: GFP-neutrophils; Blue: Sytox Blue-stained DNA; White: confocal reflection microscopy showing collagen fibers. Scale bar 50 µm. The microcolony in H is illustrated by a dotted line. The images are representative of at least three independent experiments. Data in I are averaged from three independent experiments.

In the absence of fibrinogen (3D-CoG), neutrophils migrated towards and invaded bacterial clusters of strain Newman, followed by immediate phagocytosis of staphylococci (Fig. 7A). A high ratio of these neutrophils lost their fluorescence and their nuclei became stainable by Sytox Blue, a cytoplasmic membrane impermeable fluorescent dye, indicating necrotic neutrophils.

In contrast to that, neutrophils were not able to approach and contact strain Newman microcolonies grown in 3D-CoG/Fib (Fig. 7C, D and Video S3). In order to visualize the dynamics of neutrophil migration, all single time frames of this image sequence were projected onto one another, producing a time projection (Fig. 7E). The microcolonies exhibited a halo (101+/−46 µm) free of neutrophils during the observation period of ≥3 h after neutrophil challenge. The Newman *sae* mutant microcolony which neither formed pseudocapsule nor MAM in 3D-CoG/Fib, was immediately invaded by neutrophils (Fig. 7B, Video S4 and Fig. S5), similarly to that of strain Newman in the absence of fibrinogen (Fig. 7A). The microcolonies of *eap* and *coa* mutant strains grown in 3D-CoG/Fib appeared to be protected from neutrophils similarly as strain Newman (Fig. 7I). In contrast to this, microcolonies of the Newman *vWbp emp* double mutant did not exhibit such a neutrophil-free halo, instead the neutrophils were able to reach the pseudocapsule surrounding the microcolonies (Fig. 7F–I and Video S5). Thus, the presence of the neutrophil-free halo correlated with the presence of the vWbp-dependent MAM surrounding bacterial microcolonies. Obviously, the MAM functioned as a mechanical barrier inhibiting neutrophil immigration into this zone. A possible artifact resulting from the combination of murine neutrophils with human fibrinogen could be ruled out by reproducing the migration restriction for human neutrophils isolated from peripheral blood (data not shown).

Taken together, vWbp-producing staphylococci grown in 3D-CoG/Fib produce a MAM surrounding the microcolonies which inhibits the migration of neutrophils, thus acting as a mechanical barrier.

### The Pseudocapsule Acts as a Second Mechanical Barrier against Neutrophils

**Figure 8:**
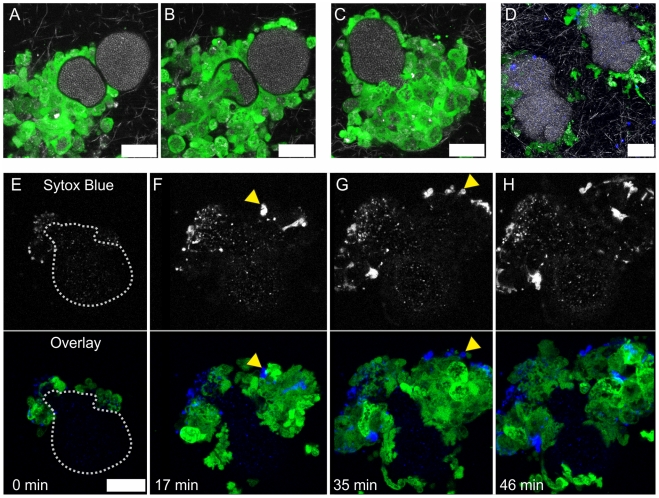
The pseudocapsule is an additional protective barrier. The pseudocapsules of *vWbp emp* double mutant microcolonies were protective against direct invasion of neutrophils into the microcolony (A, single frame from Video S6, 5 h after neutrophil challenge). After punctual rupture of the pseudocapsule, phagocytosis is initiated (B, single frame from Video S6, 27 min after A). After pseudocapsule rupture, phagocytosis is a rapid event (C, single frame from Video S7, 5 h after neutrophil challenge). USA300 barriers fulfill similar functions (D, single frame from Video S15, 3.5–5 h after neutrophil challenge). Direct contact of staphylococci with neutrophils led to massive neutrophil cell lysis/necrosis, visualized by Sytox Blue staining (yellow arrowheads, E–H, single frames from Video S8, 5 h after neutrophil challenge). In E, the microcolony outline is illustrated by a dotted line. Green: GFP-neutrophils; Blue: Sytox Blue-stained DNA; White: confocal reflection microscopy showing collagen fibers. Scale bar 20 µm.

In order to assess whether the pseudocapsule also contributed to shielding from neutrophils, we used the Newman *vWbp emp* mutant strain which was unable of MAM formation but still produced a pseudocapsule (Fig. 7F–I). As shown in Fig. 8A, immigrating neutrophils were not able to directly contact staphylococci of the microcolony. Instead, they were kept at a short distance, as can be seen from the narrow gap between neutrophils and the staphylococcal fluorescence signal in single *z* sections (see also Video S6). The dimension of this gap roughly equaled the dimension of the pseudocapsule (compare Fig. 2A and 7I). From this we suggest that the pseudocapsule acts as a second mechanical barrier sequestering the bacterial microcolonies from phagocytic cell attack. However, after about 4 h microcolony-associated neutrophils started to penetrate the pseudocapsule and to take up staphylococci. Typically, invasion of the pseudocapsule started with a localized invasive event, that is, only a single or few neutrophils squeezed through a narrow hole in the pseudocapsule. Once in direct contact with staphylococci, they immediately started phagocytosis (Fig. 8B and Video S6). This initial breakthrough of neutrophils resulted in recruitment of a wave of neutrophils and disruption of the entire colony. With some microcolonies we observed that initially a larger fragment of the pseudocapsule ruptured, allowing access of a number of neutrophils to the staphylococci. This led to a rapid dispersal of staphylococci from the ruptured pseudocapsule, while massive phagocytosis was observed (Fig. 8C and Video S7). Interestingly, phagocytosis of staphylococci after pseudocapsule rupture was frequently associated with neutrophil cell lysis/necrosis (Fig. 8E–H and Video S8). This was in agreement with neutrophil/microcolony-interaction of strain Newman grown in 3D-CoG (Fig. 7A).

In the very rare case that strain Newman wildtype or *coa* mutant microcolonies were encountered by neutrophils which had penetrated the disrupted MAM, similar events were observed (data not shown). Such mechanical injury can be occasionally observed when overlaying the 3D-CoG with a spleen slice. In the case of the *coa* mutant strain, the more irregularly shaped pseudocapsule retained residual barrier function (Video S9).

Taken together, the pseudocapsule acts as a second mechanical barrier protecting staphylococcal microcolonies from neutrophil attack.

### Pseudocapsule and MAM Barrier Function of Clinical Isolates

As shown above, formation of both a pseudocapsule and a MAM is not a unique capacity of strain Newman as it is also observed with other clinical isolates. Upon challenge with neutrophils, some isolates producing MAM were protected from neutrophil encounter (Video S10 and S11). Microcolonies of strains producing a prominent pseudocapsule but no visible MAM exhibited protection from initial neutrophil encounter but were subsequently invaded eventually, similar to the Newman *vwbp emp* mutant (Video S12 – S14). USA300 microcolonies were more accessible to neutrophils compared to strain Newman, probably due to a weaker MAM barrier function. However, upon first contact with neutrophils, barrier function similar to other clinical isolates was obvious (Fig. 8D and Video S15).

Thus, clinical isolates were able to exploit similar strategies as strain Newman in 3D-CoG/Fib to protect themselves from neutrophils.

### A Therapeutic Anticoagulant Antagonizes Staphylococcal Barrier Activity

Several synthetic thrombin protease inhibitors in clinical use have been shown to inhibit *S. aureus* coagulase activity (dabigatran [28]; argatroban [29], [30]), but inhibition of vWbp-mediated clotting has not been addressed yet. Therefore, we investigated whether argatroban could interfere with Coa-mediated pseudocapsule and vWbp-dependent MAM formation.

**Figure 9:**
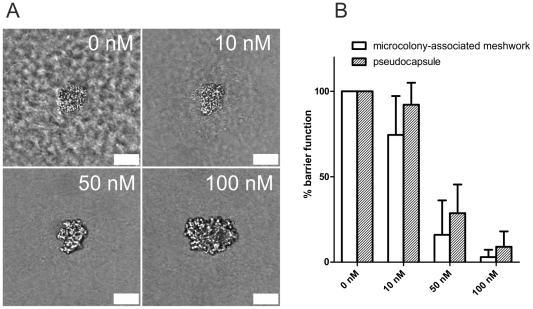
The thrombin inhibitor argatroban antagonizes staphylococcal barrier activity. *S. aureus* Newman was grown in 3D-CoG/Fib in the presence of different argatroban concentrations. After 16 h of growth, the growth phenotypes were analyzed: at 10 nM argatroban, the MAM was diminished and at higher concentrations also pseudocapsule formation was absent (A). Challenging the system with neutrophils after 16–17 h revealed loss of both barrier functions in a concentration-dependent manner (B). Scale bar 25 µm. The images are representative of three independent experiments. Data are averaged from three independent experiments.

By supplementing the growth medium (3D-CoG/Fib) with 10 nM argatroban, MAM formation was affected (Fig. 9A). Marked impairment of pseudocapsule formation required about 50 nM argatroban. These results demonstrate that argatroban is able to prevent the formation of the outer and the inner barrier generated by staphylococci grown in 3D-CoG/Fib. In consequence, microcolonies became prone to neutrophil attack: at 10 nM argatroban, 75% of the microcolonies were protected from neutrophil attack by the MAM, at 50 nM only 16% retained this barrier function, at 100 nM all microcolonies were accessible to neutrophils (Fig. 9B). Pseudocapsule function was diminished in the same argatroban concentration-dependent manner.

Thus, argatroban inhibits both pseudocapsule and MAM formation, probably by inhibiting the proteolytic activity of Coa- and vWbp-activated prothrombin. This inhibitory effect supports the disruption of microcolonies by neutrophils.

## Discussion

Here we report for the first time on growth behavior of *S. aureus* in a 3D-CoG setup supplemented with fibrinogen as a surrogate of host tissue environment. When *S. aureus* Newman is cultivated in liquid cell culture medium RPMI 1640 without agitation, bacterial clusters of variable size are formed. This is independent from the gene products of *ica*, *eap*, *emp*, *vWbp* or *coa*. Similar growth behavior of *S. aureus* was observed in 3D-CoG. The bacterial clusters were somewhat more compact due to spatial restriction by collagen fibers. Definite attachment of single staphylococci to collagen fibers was not observed. This can be explained by the fact that strain Newman lacks the collagen binding adhesin CNA [31]. From this we suggest that the 3D-CoG serves as an almost inert fibrillar collagen meshwork for strain Newman. Consequently the 3D-CoG can be used as a migration substrate for neutrophils when studying staphylococci-neutrophil interactions.

A characteristic feature of *S. aureus* is its capability to convert fibrinogen into fibrin by activating prothrombin via the secreted proteins Coa and vWbp. Therefore we added human fibrinogen to our assay (3D-CoG/Fib). Under these conditions, strain Newman forms regular microcolonies which are surrounded by two concentric structures: an inner spherical pseudocapsule and an outer dense microcolony-associated meshwork (MAM). Both obviously contain fibrin as they can be degraded by plasmin. This architecture resembles that of staphylococcal abscess communities (SAC) in experimental murine infection described by Cheng *et al.* [14], [15] and Sawai *et al.* [32]. We could show that the staphylococcal clotting factors Coa and vWbp are required for the formation of these structures. Firstly, Coa was detected in association with the pseudocapsule by immunostaining. This suggests that Coa is retained and accumulated in the vicinity of the pseudocapsule and activates prothrombin. Furthermore, we demonstrated that a *coa* mutant strain forms more irregularly shaped pseudocapsules. This seems to influence the shape of the microcolony.

Secondly, formation of the MAM is not affected in a *coa* mutant but completely abolished in a *vWbp* mutant. From this, we conclude that vWbp, and not Coa, is the clotting factor involved in MAM formation. Assuming that the *coa* mutant is producing only vWbp as clotting factor, we can conclude that vWbp is also able to partially take over Coa function by supporting partial formation of the pseudocapsule.

**Figure 10:**
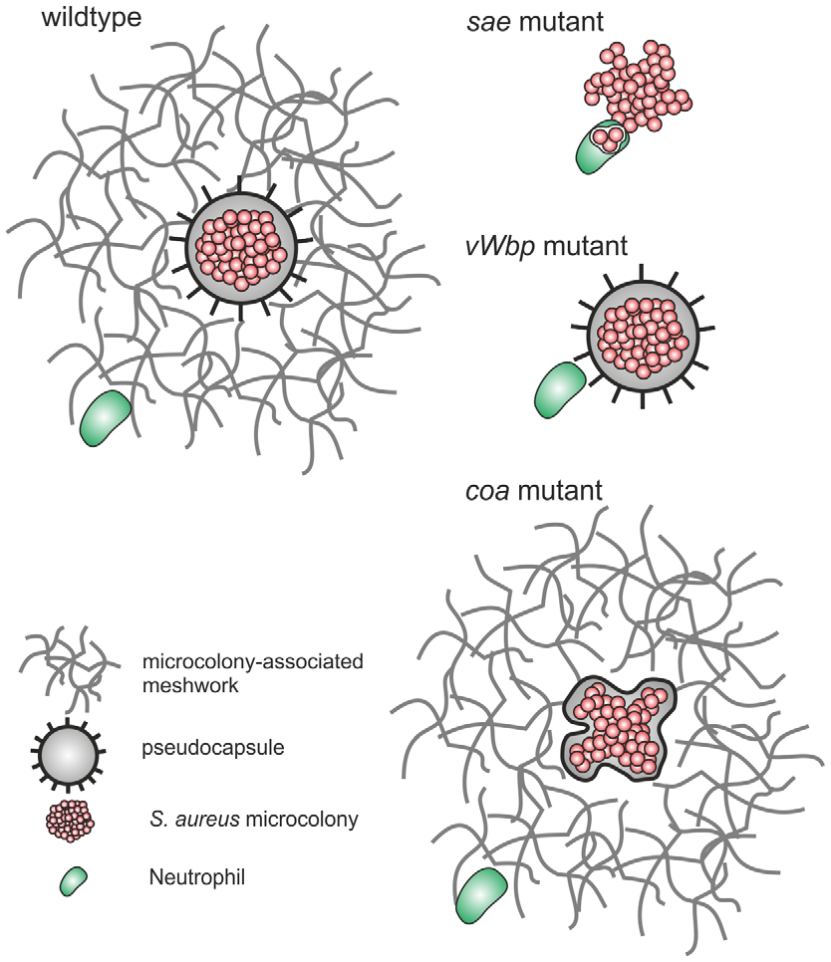
Proposed model for the differential actions of Coa and vWbp. *S. aureus* Newman forms two concentric barriers in the presence of fibrinogen in the growth medium: the outer MAM and the inner pseudocapsule; both contain fibrin. The MAM is dependent on vWbp and inhibits neutrophil immigration into the vicinity of the microcolony. The pseudocapsule is partially dependent on Coa and acts as a second barrier preventing neutrophil invasion of the microcolony.

From these results we propose the following model (Fig. 10): single *S. aureus* cells give rise to microcolonies during growth in 3D-CoG/Fib. Staphylococcal clotting factors Coa and vWbp mediate conversion of soluble fibrinogen to insoluble fibrin. This leads to fibrin deposition in the vicinity of the microcolony, resulting in two independent structures: an inner pseudocapsule and an outer MAM. Eap and Emp are apparently not required for the formation of these microscopically visible structures, though Emp localized on or within the pseudocapsule in a similar manner to Coa.

It is of note that both Coa and vWbp have no direct proteolytic activity for fibrinogen conversion but they hijack the host clotting machinery by activating prothrombin independently of the coagulation cascade [9], [11]. Traces of prothrombin, plasminogen and vWF can be present in commercially available fibrinogen prepared from human plasma. Obviously, even these prothrombin traces appear to be sufficient for the observed clotting activity of *S. aureus* in 3D-CoG. However, during our experiments we encountered a fibrinogen batch which did not induce the described encapsulation. In that case addition of 2–5 µg/ml prothrombin to 3D-CoG/Fib restored the phenotype (data not shown).

Both Coa and vWbp are members of the bifunctional zymogen activator and adhesion protein (ZAAP) family: besides their prothrombin-binding and -activating function, they possess binding properties for other host proteins such as fibrinogen and vWF [10]. Moreover, vWF is capable of binding collagen fibers [33]. It has been discussed that such binding activities might be responsible for localized coagulation activity by acting as a homing device to direct these proteins to a certain spatial context after release from staphylococci. Our results support this hypothesis by spatial dissection of the clotting activity due to the formation of two discrete capsule-like structures. This is further corroborated by a recent study in a mouse infection model which reports that Coa is localized to a pseudocapsule enclosing the staphylococci, whereas vWbp was also found more distant in the abscesses [15]. Therefore we suggest that Coa activity might be restricted to a narrow zone of the microcolony interface, probably by binding to a released staphylococcal protein or via a host-derived bridging molecule. From this localization it might exert a short-distance effect on fibrinogen conversion, resulting in pseudocapsule formation. In contrast, vWbp acts preferentially in the periphery of the microcolony, mediating the formation of the MAM (long-distance effect).

These conclusions are supported by the growth behavior of the *saeRS*-mutant which is impaired in secretion of Coa and vWbp ([34], [35], compare Fig. S1) and thus is unable to form the MAM or the pseudocapsule.

Another important virulence mechanism of *S. aureus* is its ability to degrade fibrin clots which play a role in sequestering infected foci from healthy tissue in abscesses. This is suggested to lead to dissemination of staphylococci into deeper and more remote tissue. After extended periods of growth (20–40 h), we detected single microcolonies which exhibited massive growth and subsequent dispersal probably after disintegration of the pseudocapsule and MAM. *S. aureus* is able to induce fibrin degradation by activation of host plasminogen by staphylokinase [36]. Staphylokinase secretion is positively regulated by the *agr* system [37] and its production peaks in stationary phase [38]. We showed that the observed occasional dispersal of microcolonies after incubation for extended periods of time is affected in an *agr* mutant and therefore suggest that staphylokinase might play a role. However, staphylokinase requires plasminogen for conversion into plasmin which in turn degrades fibrin. Commercially available fibrinogen purified from human plasma is known to contain traces of plasminogen. The inhibition of fibrinolysis by the serine protease inhibitor aprotinin or by the specific plasmin inhibitor tranexamic acid further corroborates the involvement of plasmin-staphylokinase. Of note, both compounds did not affect pseudocapsule or MAM formation.

Only recently, the concept of *S. aureus* hijacking the host clotting machinery to establish a protective niche has gained support from experimental mouse infection [14], [15]. In corroboration of this concept, we asked whether the observed two discrete fibrin capsule-like structures mediate any barrier function for immigrating phagocytes and whether pseudocapsule and MAM function differently in regard of phagocyte invasion.

Neutrophils are considered to be the first line of cellular defense of localized infections. Moreover, their predominance in abscesses formed by staphylococci has been shown [14]. Here, we have established for the first time an *in vitro* approach to study neutrophil-staphylococci interactions in 3D-CoG/Fib by using native spleen slices as a source of neutrophils. This approach has advantages in comparison to the application of neutrophils isolated from blood or bone marrow: Firstly, the neutrophils are not affected by various steps during the isolation procedure. Moreover, their inherent ability of amoeboid migration in 3D-CoG serves as a coarse passive phenotypical isolation step. Secondly, spleen slices provide a steady supply of native murine neutrophils throughout the experiments by acting as “neutrophil-soaked sponges”. Compared to overlaying the 3D-CoG with blood only or neutrophils in solution, we observed more consistent neutrophil migration into the 3D-CoG (data not shown). This might be explained by facilitating the entry of neutrophils into the pre-formed 3D-CoG by supplying a direct and close tissue/3D-CoG contact instead of a liquid/3D-CoG interface. However, this model can be used with isolated neutrophils from other sources as well (e. g. neutrophils isolated from peripheral blood, dHL-60 cell line). Thirdly, neutrophils from various transgenic or knock out mouse strains can be used in this system in order to assess the impact of gene deletions on specific host-pathogen interactions. Such studies are currently conducted in our laboratory.

As shown previously, neutrophils migrate randomly in 3D-CoG [39]. In this study we observed unrestricted neutrophil migration towards *S. aureus* microcolonies pre-grown in 3D-CoG, followed by high rates of phagocytosis. However, in the presence of fibrinogen, we observed that the vWbp-mediated MAM probably acted as a mechanical barrier and prevented neutrophil migration towards the staphylococcal microcolony. In addition to mechanical restraints imposed onto neutrophils, it is also conceivable that released *S. aureus* proteins bind to the fibrin meshwork and interfere with neutrophil signaling pathways involved in chemotaxis. This remains to be elucidated.

Strikingly, the pseudocapsule turned out to be a second safety barrier against neutrophil attack. Only after accumulation of a higher number of neutrophils at the interface of the microcolony we observed selected neutrophils being able to penetrate. This might be due to a bacteria-mediated dispersal mechanism degrading the pseudocapsule or more likely due to direct degradation of the pseudocapsule by released neutrophil proteases. These neutrophils obviously gained entry to the microcolony by squeezing through small holes. The penetration of such a “pioneer neutrophil” elicited a massive attraction and invasion of neutrophils to the interior of the microcolony. Whether pioneer neutrophils release chemokines or whether destruction of the pseudocapsule leads to the release of staphylococcal chemoattractants remains to be elucidated.

Interestingly, we observed a high rate of neutrophil cell lysis/necrosis after direct contact of neutrophils with staphylococci regardless of whether microcolonies were previously surrounded by a pseudocapsule or not. Probably neutrophils are killed after phagocytosis and oxidative burst or by toxic substances released by staphylococci. It is conceivable that staphylococci reached stationary phase after pseudocapsule formation and start toxin production, resulting in “caged toxins”. By using mutants affected in toxin production (e. g. PSMs or α-hemolysin), it should be possible to unravel the mechanism of this staphylococci-induced neutrophil cell death after pseudocapsule rupture. Of note, it has been reported recently that CA-MRSA strains induce a form of programmed necrosis [40].

Taken together, both the pseudocapsule and the MAM exert a strong barrier function for neutrophils and protect staphylococci against phagocytosis. However, compared to the rapid phagocytosis of staphylococci after barrier breakdown, this suggests that other reported anti-phagocytosis activities, e.g. ClfA-mediated phagocytosis inhibition [41], are minimal compared to this massive phagocytosis inhibition. Various studies have shown that strain Newman differs from other *S. aureus* model strains, mainly due to a missense mutation in *saeS* resulting in derepression of gene expression [13]. In order to check, whether the results obtained with strain Newman are unique, we compared several clinical *S. aureus* isolates and found similar characteristics. Presence of a pseudocapsule appears to be a very common phenomenon, while formation of MAM showed strong variation among the analyzed isolates. This is in line with the reported pseudocapsule of strain USA300 in the mouse infection model [14].

Several thrombin inhibitors have been reported to inhibit Coa-mediated activation of prothrombin [28], [30]. We wondered if these agents have an impact on the growth phenotypes of *S. aureus* in 3D-CoG/Fib and on the barrier function for neutrophils. Indeed, argatroban, a thrombin inhibitor in clinical use, prevented pseudocapsule and MAM formation in a concentration-dependent manner and by this enhanced neutrophil access to staphylococci.

This result provides us with an attractive therapeutic option in combating staphylococcal infections: By hijacking host machinery and relying on this mechanism for virulence, *S. aureus* offers a specific host-derived target. This target is well characterized, not least for its importance in coagulation-associated diseases. Early access of neutrophils to staphylococci can be expected to counteract this virulence advantage gained by usurping host machinery. These results may lead to new strategies in treatment of staphylococcal infections by using protease inhibitors in combination with antibiotics.

Furthermore, it should be mentioned that a species specificity for various clotting-related *S. aureus* proteins has been shown: staphylokinase shows high activity towards human but only limited activity towards murine plasminogen [38], coagulase activates bovine and rabbit prothrombin only weakly compared to human prothrombin [17], and recently it has been shown, that certain *S. aureus* strains carry species-specific *vWbp* alleles [42]. Thus, the presented 3D-CoG infection model can be useful in the study of such host specificities by supplementing the system with further plasma proteins, as well as with additional cell types, e. g. mesenchymal cells.

Nevertheless, it has to be stated that this is a reductionstic *in vitro* model lacking the complexity of an *in vivo* infection model. However, it will be helpful in improving the design of animal infection experiments and to complement the interpretation of *in vivo* results.

Taken together, the 3D-CoG model in combination with native spleen slices is a suitable *in vitro* infection model to study both *in vivo*-like growth characteristics and the resulting phagocyte-microbe interactions. It opens a broad field of applications by complementing established animal infection models for the development of new treatment options for infections.

## Materials and Methods

### Ethics Statement

All experimental procedures involving animals were performed according to the “German Animal Protection Act” (TierSchG) and approved by the regional authorities of the city of Munich (KVR-I/221, TA077/10). Human serum was pooled from voluntary donors at the Max von Pettenkofer-Institute, LMU Munich, Germany, according to approval by the ethics commission of the Medical Faculty of the LMU Munich. Written, informed consent was provided by the volunteers. Clinical *S. aureus* isolates were obtained as discarded de-identified isolates from the clinical microbiological laboratory of the University Hospital of Munich.

### Strains and Growth Conditions

The strains and plasmids used in this study are listed in Table 1 and Table 2. Staphylococci were routinely cultured under constant agitation in LB or Basic medium (BM; 1% peptone, 0.5% yeast extract, 0.1% glucose, 0.5%NaCl, 0.1% K_2_HPO_4_) supplemented with antibiotics if the strains carry resistance cassettes (50 µg/ml kanamycin, 10 µg/ml erythromycin, 5 µg/ml tetracycline, 10 µg/ml chloramphenicol, 100 µg/ml ampicillin). RPMI 1640 medium (No Phenol Red, Invitrogen) was used as a growth medium for 3D-CoG.

**Table 1:** Strains used in this study.

Strain	Properties	Reference
Newman	wildtype strain	[45]
Newman-29	*ΔsaePQRS::kan*	[13]
Newman-29, 33	complementation of Newman-29	[13]
Newman *coa*	*Δcoa::tetK*	[46]
Newman *vWbp emp* *	*Δemp::erm*	[47]
Newman *eap*	*Δeap::erm*	[43]
Newman *agr* (ALC355)	*Δagr::tetM*	[48]
MP1-11	Clinical MSSA isolate, blood culture	Obtained from University Hospital LMU Munich
MP2-11	Clinical MSSA isolate, blood culture	Obtained from University hospital LMU Munich
MP3-11	Clinical MSSA isolate, blood culture	Obtained from University Hospital LMU Munich
MP6-11	Clinical MSSA isolate, respiratory tract	Obtained from University Hospital LMU Munich
MP9-11	Clinical MSSA isolate, abscess sample	Obtained from University Hospital LMU Munich
MP10-11	Clinical MRSA isolate, abscess sample	Obtained from University Hospital LMU Munich
USA300 FPR3757	CA-MRSA strain	[24]
ST239-CC8	MRSA strain	[25]
RN4220	restriction-deficient *S. aureus* strain	[49]
DH5α	*E. coli* strain for plasmid modifications	[50]

*Formerly denoted as Newman *emp* [47]. Sequencing analysis revealed that assumedly by phage transduction not only the *emp* gene but also the neighboring functional *vWbp* gene of strain Newman has been replaced by a truncated version of *vWbp* from strain RN4220. This was further confirmed by the fact that vWbp could be detected in the supernatants of 3 h Newman wildtype cultures but not of this *emp* mutant strain (Fig. S1).

**Table 2:** Plasmids used in this study.

Plasmid	Properties	Reference
pSK236	Shuttle vector containing pUC19 cloned into the *HindIII* site of pC194	[51]
pEmp	a 1.3 kb fragment containing the *emp* gene from strain Newman was cloned into pSK236 (*BamHI*, *PstI*)	This study
pvWbp	a 1.9 kb fragment containing the *vWbp* gene from strain Newman was cloned into pSK236 (*EcoRI*, *SalI*)	This study
pvWbpEmp	a 1.9 kb fragment containing the *vWbp* gene (*EcoRI*, *BamHI*) and a 1.3 kb fragment containing the *emp* gene (*BamHI*, *PstI*) from strain Newman were cloned into pSK236	This study

### Construction of Complementing Plasmids

The *vWbp* gene and the *emp* gene were amplified from chromosomal DNA of strain Newman by PCR (High Fidelity PCR Enzyme Mix, Fermentas) using the primer pairs vWbp-f-EcoRI/vWbp-r-SalI (1), vWbp-f-EcoRI/vwb-r-BamHI (2) and emp-f-BamHI/emp-r-PstI (3), respectively (Table S1). The PCR products (1) and (3) were digested with EcoRI/SalI and BamHI/PstI, respectively, and ligated into pSK236 isolated from DH5α digested in the same way, resulting in the plasmids pvWbp and pEmp. In order to produce pvWbpEmp, the PCR product from (2) was cloned into pSK236 via EcoRI/BamHI, followed by cloning of the PCR product from (3) into the resulting plasmid. Ligation products were transformed into electrocompetent DH5α. Transformants were screened for growth with ampicillin. The resulting plasmids were isolated from DH5α and transformed into electrocompetent RN4220, isolated again and then transformed into electrocompetent Newman wildtype or *vWbp emp* mutant strain.

### Analysis of Protein Secretion

Presence of Emp on the *S. aureus* cell surface was detected as described previously with some modifications [43]: *S. aureus* was cultivated in 10 ml of BM medium for 18 h. Differences in optical density at 600 nm (OD600) were adjusted by addition of BM medium. Cells were harvested by centrifugation at 4,000 x g for 10 min. The pellets were resuspended in 300 µl of 2% sodium dodecyl sulfate (SDS, Sigma) and incubated at 95°C for 5 min shaking at 750 rpm. The supernatant was then isolated in two sequential centrifugation steps at 10,000 x g for 5 min and stored at −80°C. Equal amounts were mixed with sample buffer and proteins were separated by 12% SDS-polyacrylamide gel electrophoresis (PAGE).

For analysis of secreted proteins, overnight cultures were diluted 1∶25 in 5 ml of fresh LB medium. After 3 h of growth, secreted proteins were precipitated from 3.6 ml culture supernatants with 10% trichloroacetic acid for 1h on ice. The pellets were washed three times in −20°C aceton (12.000 x g, 10 min, 4°C), followed by a final washing step in H_2_O. The pellet was dried and resuspended in sample buffer. Equal amounts of supernatant according to the OD600 before harvesting were separated by 10% SDS-PAGE.

### 3D-CoG Infection Model

Collagen gels (3D-CoG) were generated as described previously [21] and incubated without agitation. Staphylococci were grown in LB medium with agitation before inoculation into 3D-CoG and subsequently suspended in liquid collagen solution (about 2×10^4^/ml). Liquid collagen solution consisted of 1.78 mg/ml bovine type I collagen (Purecol, Advanced Biomatrix) in RPMI 1640 medium adjusted to pH 7.4. 10 µl of this solution were dispersed on the bottom of a microscopic dish (9.4×10.7 mm, µ-slide 8 well, ibidi). The samples were allowed to polymerize for 45 min (37°C; 5% CO_2_). 3D-CoGs were overlaid with 150 µl medium: RPMI 1640 or RPMI 1640 supplemented with 3 mg/ml fibrinogen from human plasma (Calbiochem) (3D-CoG/Fib). For protease inhibition experiments, the plasmin inhibitors aprotinin and tranexamic acid or the thrombin inhibitor argatroban were obtained from Santa Cruz and added directly to the growth medium.

For neutrophil challenge of staphylococcal microcolonies in 3D-CoG, native spleens from 8–12 weeks old heterozygous *lys-EGFP* C57BL/6 mice [27] were used. *Lys-EGFP* mice were bred and maintained at the Max von Pettenkofer-Institute in isolated ventilated cages (Tecniplast) under SPF conditions. All animal work was conducted according to the relevant national and international guidelines. Mice were sacrificed by CO_2_ asphyxiation and spleens were harvested and cut into 300 µm slices with a vibrating blade microtome (Leica) at 4°C. Supernatants from 3D-CoG samples were removed and then 3D-CoG were overlaid with spleen slices. To immobilize the slices on the 3D-CoG, a drop of 4% NuSieve GTG agarose (Lonza) was applied. After solidification, 150 µl of RPMI 1640 were added. Sytox Blue for staining of cells with corrupted membranes was used at 1 µM according to the manufacturer's protocol (Invitrogen). The manual process of overlaying 3D-CoG with tissue slices can partially result in compression or injury of some areas of the collagen gel. To compensate for artifacts, only microcolonies in a lateral distance of about 200 µm to the tissue slice-collagen gel interface were analyzed after verifying that the collagen gel in this area was not compressed or injured. This was achieved by visualization of the 3D-CoG structure with confocal reflection microscopy. The samples were incubated at 37°C/5% CO_2_ in a cell culture incubator or in the microscope incubation chamber.

### Microscopy and Image Processing

Confocal laser scanning microscopy (CLSM) was performed on a Leica SP5 microscope (Leica, Germany) equipped with an incubation chamber (The Cube & The Box, Life Imaging Services, Switzerland). Images were acquired with a 63x oil immersion objective, a 40x oil immersion objective or a 10x objective. Image acquisition, processing and quantification were performed with LAS AF software (Leica, Germany).

Confocal reflection contrast microscopy was used to visualize unstained collagen and fibrin fibers as described previously [44]. The dimensions of z projections from *xyz* stacks are mentioned in the figure legends. Time projections of *xyzt* series were performed by projecting all single *xy* frames onto a single one.

### Immunocytochemistry

All steps were performed in a volume of 150 µl in microscopic dishes (µ-slide 8 well, ibidi) at room temperature. CoG were fixed with 4% paraformaldehyde for 20 min at room temperature and then washed three times (PBS, 5 min each). Samples were blocked for 1 h in blocking buffer (PBS with 3% bovine serum albumin and 5% human serum from donors) for Coa- or Emp-staining or with 5% goat serum in TBS-T for Ly-6G staining. Primary antibody was added to the samples (1∶100 rabbit anti-Coa, 1∶333 rabbit anti-Emp; both were kindly provided by O. Schneewind; 1∶100 anti-Ly-6G, BD Biosciences) and incubated for 1.5 h, followed by three washing steps (PBS or TBS-T, 5 min each). Secondary antibody (1∶200 Alexa Fluor 555 goat anti-rabbit or Alexa Fluor 647 goat anti-rat, Invitrogen) was diluted in blocking buffer, added to the samples and incubated for 1 h in the dark, followed by three washing steps (PBS, 5 min each). 1 µg/ml DAPI (Sigma-Aldrich) was used for staining of DNA. Cell surface of staphylococci was stained with 5 µg/ml FITC-labeled lectin from *T. vulgaris*, specific for N-Acetyl-glucosamine (Sigma-Aldrich). Additional stainings were performed in parallel to incubation with secondary antibody.

### Statistical Analysis

Statistical significance calculations were performed by Student's unpaired *t*-test.

### Accession Numbers

The GenBank (http://www.ncbi.nlm.nih.gov/genbank/) accession number for genes discussed in this paper are: Coa (5330026), vWbp (5331820), Emp (5330439).

## Supporting Information

Figure S1 **Production of Coa, vWbp and Emp.** Coa and vWbp were detected in supernatants of 3 h cultures and verified by MALDI-TOF (A). The *vWbp emp* double mutant secreted no vWbp, the *coa* mutant secreted no Coa, the *sae* mutant (New29) secreted neither detectable Coa nor vWbp. Ectopic expression of *vWbp* from pvWbp led to hypersecretion of vWbp. Emp and Eap were detected in SDS surface extracts (B). The *vWbp emp* double mutant is defective in Emp production. pEmp restored this defect and caused hypersecretion of Emp.
(TIF)

Figure S2 **vWbp is responsible for formation of the MAM.** The *vWbp emp* double mutant was complemented with plasmids encoding *emp* (pEmp) or *vWbp* (pvWbp) alone or both (pvWbpEmp) under their native promoters. This *in trans* approach led to overproduction of the respective proteins (compare Fig. S1). Expression of Emp from the plasmid caused increased size and more irregular shape of microcolonies but did not complement the lacking MAM. In contrast to this, expression of vWbp from the plasmid restored the MAM phenotype (blue arrowhead) and led to an increased diameter, possibly due to overproduction. Scale bar 150 µm. The images (A–C) are representative of three independent experiments. Data (D) are averaged from two independent experiments.
(TIF)

Figure S3 **Inhibition of fibrin degradation by plasmin inhibitors.** Aprotinin (final concentration 12,6 µM) or tranexamic acid (final concentration 3 mM) were added to the growth medium at t = 0h. This was repeated after 24h and 48h in order to compensate for possible decay of the inhibitor activity. Even after 6 days no fibrin degradation surrounding microcolonies could be observed. Three representative sections are shown. Scale bar 100 µm.
(TIF)

Figure S4 **GFP^+^ cells migrating into 3D-CoG are Ly-6G^+^.** Spleen slices from heterozygous *lys-EGFP* C57BL/6 mice were layered on top of preformed 30 µl 3D-CoG and incubated at 37°C for 4 h. Subsequently, the spleen slice was removed and cells inside the 3D-CoG were immunostained for Ly-6G. All GFP^+^ cells were Ly-6G^+^. An area including a GFP^-^ cell is selected to include a negative Ly-6G^-^ control cell. B is a magnification of the inset in A. A: scale bar 50 µm. B: scale bar 25 µm.
(TIF)

Figure S5 **Time projection of interaction of neutrophils with Newman** ***sae*** **mutant in 3D-CoG/Fib.** *S. aureus sae* mutant clusters grown in 3D-CoG/Fib for 17 h were invaded and phagocytosed by neutrophils without delay. Figure S5 shows a time projection of the entire observation period (87 min) of Video S4 (projection of two sections spanning a total depth of 5.3 µm). Green: GFP-neutrophils; White: confocal reflection microscopy showing collagen fibers. Scale bar 75 µm.
(TIF)

Table S1 **Oligonucleotides used in this study.**
(DOC)

Video S1 **Degradation of pseudocapsule and MAM by plasmin.** *S. aureus* Newman microcolonies were grown in 3D-CoG/Fib for 16 h. Subsequent addition of plasmin (8 µg/ml) to the 3D-CoG/Fib surface led to rapid degradation of both pseudocapsule and MAM surrounding Newman microcolonies within few minutes. Duration: 22 min.
(AVI)

Video S2 **Fibrinolysis of CA-MRSA strain USA300.** A single USA300 microcolony degrading pseudocapsule and MAM 21 h after inoculation in 3D-CoG/Fib (10x bacterial density compared to normal assays to provoke fibrinolysis). This is a time lapse video representing the bottom left area of Fig. 5C. Fibrin structures are degraded whereas collagen fibers are unaffected. A projection of several sections spanning a total depth of 11 µm is shown. Confocal reflection microscopy shows collagen and fibrin fibers. Duration: 140 min.
(AVI)

Video S3 **Interaction of neutrophils with** ***S. aureus*** **Newman in 3D-CoG/Fib.** *S. aureus* Newman was grown in 3D-CoG/Fib for 17 h and then challenged with neutrophils. Neutrophils migrated within the 3D-CoG but did not approach the colonies within 3 h of observation. Figure 7E shows a time projection of the entire observation period. Green: GFP-neutrophils; White: confocal reflection microscopy showing collagen fibers. A projection of 3 sections spanning a total depth of 10.3 µm is shown. Duration: 3 h.
(AVI)

Video S4 **Interaction of neutrophils with** ***S. aureus*** **Newman** ***sae*** **mutant in 3D-CoG/Fib.** Newman *sae* mutant (Newman-29) clusters grown in 3D-CoG/Fib for 17h were immediately invaded and phagocytosed by neutrophils. Figure S5 shows a time projection of the entire observation period. Green: GFP-neutrophils; White: confocal reflection microscopy showing collagen fibers. A projection of two sections spanning a total depth of 5.3 µm is shown. Duration: 87 min.
(AVI)

Video S5 **Interaction of neutrophils with** ***S. aureus*** **Newman** ***vWbp emp*** **mutant in 3D-CoG/Fib.** Newman *vWbp emp* mutant was grown in 3D-CoG/Fib for 17 h and then challenged with neutrophils. Neutrophil migration in the vicinity of the microcolony was not inhibited. Neutrophils approached the microcolony but were prevented from invading by the pseudocapsule. Figure 7H shows a time projection of the whole observation period. Green: GFP-neutrophils; White: confocal reflection microscopy showing collagen fibers. A projection of 3 sections spanning a total depth of 10.3 µm is shown. Duration: 3 h.
(AVI)

Video S6 **Protective function of the pseudocapsule.** Pseudocapsules protected *S. aureus* from direct invasion by neutrophils into the microcolony. This is shown here with the *vWbp emp* double mutant. 17 h after inoculation, bacterial microcolonies were challenged with neutrophils. After approximately 5 h 15 min of neutrophil challenge, the first neutrophils invaded the microcolony and started phagocytosis. Green: GFP-neutrophils; Blue: Sytox Blue-stained DNA; White: confocal reflection microscopy showing collagen fibers. Duration: 45 min.
(AVI)

Video S7 **Pseudocapsule rupture.** Pseudocapsules protected *S. aureus* from direct invasion by neutrophils into the microcolony. This is shown here with the *vWbp emp* double mutant. 17 h after inoculation, bacterial microcolonies were challenged with neutrophils. After approximately 5 h the pseudocapsule ruptured and released huge amounts of staphylococci which are phagocytosed by surrounding neutrophils. Green: GFP-neutrophils; Blue: Sytox Blue; White: confocal reflection microscopy. Duration: 45 min.
(AVI)

Video S8 **Neutrophil cell lysis/necrosis after pseudocapsule rupture.** Direct contact of neutrophils with staphylococci due to pseudocapsule rupture or invasion led to massive neutrophil cell lysis/necrosis, visualized by Sytox Blue staining of nuclei. The *vWbp emp* double mutant was grown in 3D-CoG/Fib for 17 h and then challenged with neutrophils. 5 h later this video was taken. Green: GFP-neutrophils; Blue: Sytox Blue-stained DNA; White: confocal reflection microscopy showing collagen fibers. Duration: 45 min.
(AVI)

Video S9 **The irregularly shaped pseudocapsule of** ***coa*** **mutant retains residual barrier function.** The *coa* mutant was grown in 3D-CoG/Fib for 17 h and then challenged with neutrophils. 3 h later this video was taken. Residual barrier function of the irregularly shaped pseudocapsule could be detected in some sections of the microcolony. Green: GFP-neutrophils; Blue: Sytox Blue-stained DNA; White: confocal reflection microscopy showing collagen fibers. Duration: 35 min.
(AVI)

Video S10 **Interaction of neutrophils with MSSA MP2-11 in 3D-CoG/Fib.** MP2-11 was grown in 3D-CoG/Fib for 17 h and then challenged with neutrophils. 1 h later this video was taken. The microcolonies before neutrophil challenge are shown in Fig. 4F. The MAM-like structure appears to be a barrier for neutrophil migration. Green: GFP-neutrophils; White: confocal reflection microscopy showing collagen fibers and staphylococci. A projection of 3 sections spanning a total depth of 17 µm is shown. Duration: 2 h 15 min.
(AVI)

Video S11 **Interaction of neutrophils with MRSA ST239-CC8 in 3D-CoG/Fib.** ST239-CC8 was grown in 3D-CoG/Fib for 17 h and then challenged with neutrophils. 1.5 h later this video was taken. The MAM-like structure appears to be a barrier for neutrophil migration. Green: GFP-neutrophils; Blue: Sytox Blue-stained DNA; White: confocal reflection microscopy showing collagen fibers and staphylococci. A single plane is shown. Duration: 65 min.
(AVI)

Video S12 **Interaction of neutrophils with MSSA MP9-11 in 3D-CoG/Fib.** MP9-11 was grown in 3D-CoG/Fib for 17 h and then challenged with neutrophils. 1 h later this video was taken. Unrestricted neutrophil migration in the periphery of the microcolony can be seen as well as barrier function of the pseudocapsule and subsequent invasion. Green: GFP-neutrophils; White: confocal reflection microscopy showing collagen fibers and staphylococci. A projection of 3 sections spanning a total depth of 9 µm is shown. Duration: 2 h 15 min.
(AVI)

Video S13 **Interaction of neutrophils with MSSA MP3-11 in 3D-CoG/Fib.** MP3-11 was grown in 3D-CoG/Fib for 17 h and then challenged with neutrophils. 1 h later this video was taken. Unrestricted neutrophil migration in the periphery of the microcolony can be seen as well as barrier function of the pseudocapsule during microcolony growth. Green: GFP-neutrophils; White: confocal reflection microscopy showing collagen fibers and staphylococci. A projection of 3 sections spanning a total depth of 15 µm is shown. Duration: 2 h 15 min.
(AVI)

Video S14 **Interaction of neutrophils with MSSA MP6-11 in 3D-CoG/Fib.** MP6-11 was grown in 3D-CoG/Fib for 17 h and then challenged with neutrophils. 1 h later this video was taken. Unrestricted neutrophil migration in the periphery of the microcolony can be seen as well as barrier function of the pseudocapsule and subsequent destruction of the entire microcolony. Green: GFP-neutrophils; White: confocal reflection microscopy showing collagen fibers and staphylococci. A projection of 3 sections spanning a total depth of 13 µm is shown. Duration: 2 h 15 min.
(AVI)

Video S15 **Interaction of neutrophils with MRSA USA300 in 3D-CoG/Fib.** USA300 was grown in 3D-CoG/Fib for 17 h and then challenged with neutrophils. 3.5 h later this video was taken. Certain barrier function in some areas of the microcolony of MAM/pseudocapsule could be demonstrated (lower part), whereas neutrophils take up staphylococci in areas of dispersal (left microcolony, upper part). Green: GFP-neutrophils; Blue: Sytox Blue-stained DNA; White: confocal reflection microscopy showing collagen fibers. Duration: 87 min.
(AVI)
