# Supplementary figures and images for: Two Distinct Coagulase-Dependent Barriers Protect Staphylococcus aureus from Neutrophils in a Three Dimensional in vitro Infection Model
Source: PLoS Pathog. 2012 Jan 12;8(1):e1002434. doi: 10.1371/journal.ppat.1002434 (PMC3257306; doi:10.1371/journal.ppat.1002434)

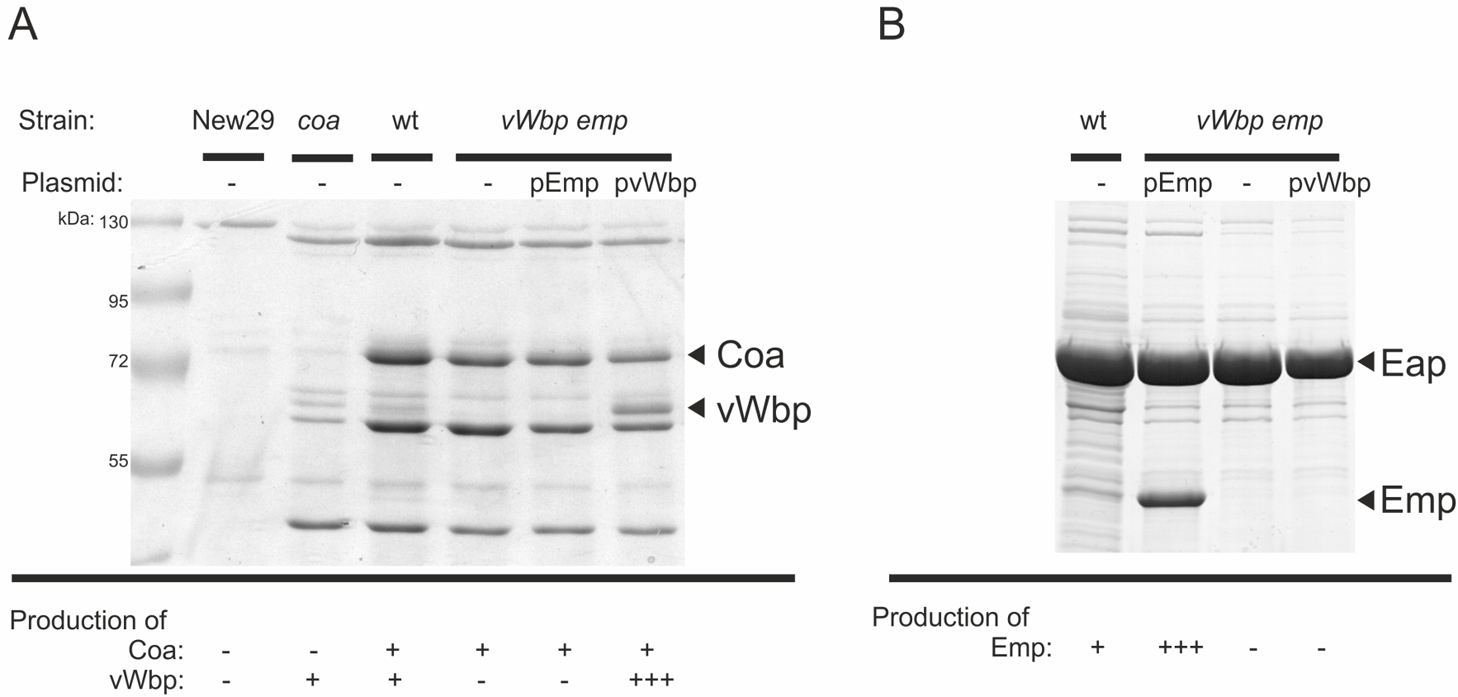

Supplement: Figure S1 — Production of Coa, vWbp and Emp. Coa and vWbp were detected in supernatants of 3 h cultures and verified by MALDI-TOF (A). The vWbp emp double mutant secreted no vWbp, the coa mutant secreted no Coa, the sae mutant (New29) secreted neither detectable Coa nor vWbp. Ectopic expression of vWbp from pvWbp led to hypersecretion of vWbp. Emp and Eap were detected in SDS surface extracts (B). The vWbp emp double mutant is defective in Emp production. pEmp restored this defect and caused hypersecretion of Emp. (TIF) [file ppat.1002434.s001.tif]

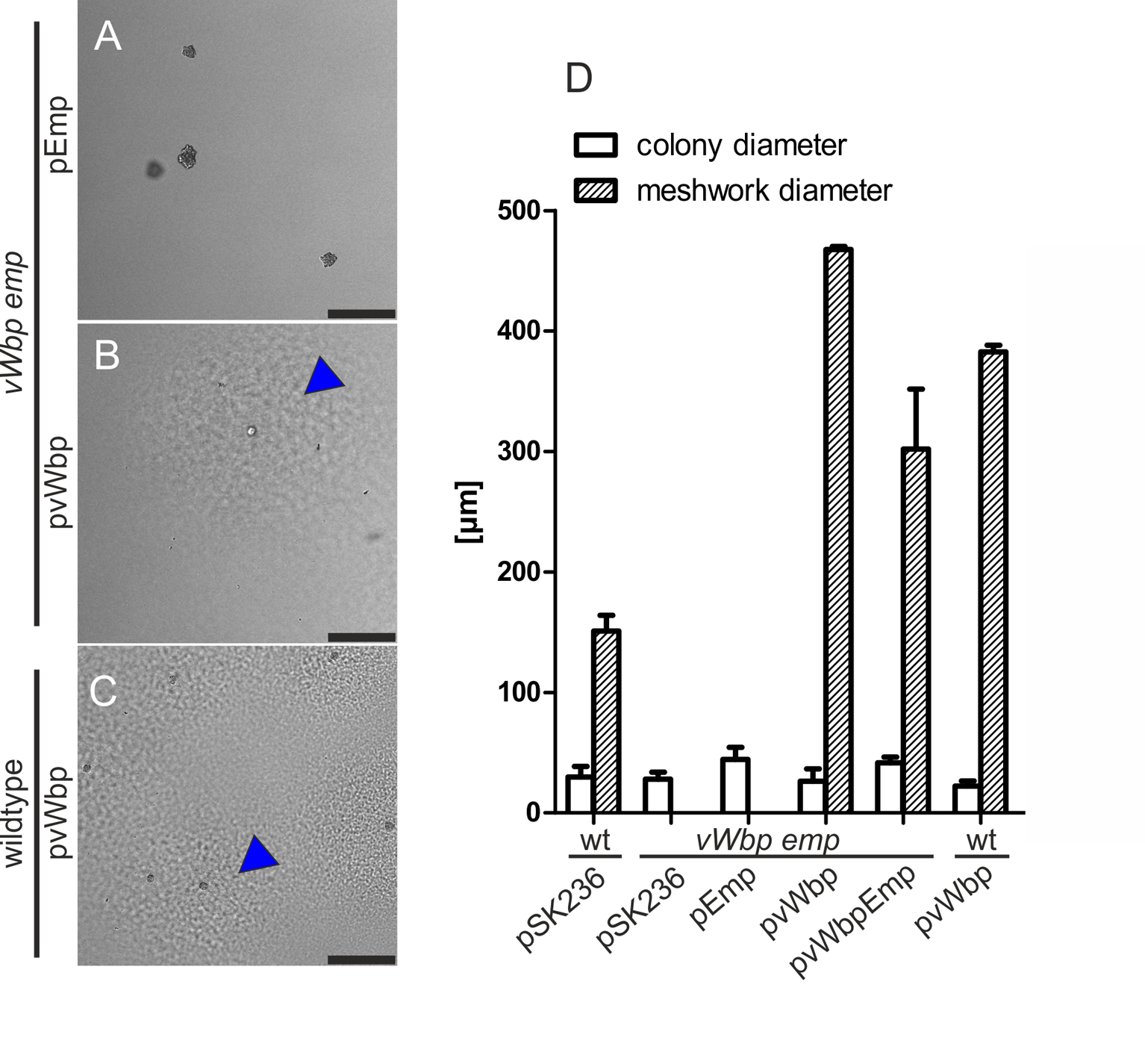

Supplement: Figure S2 — vWbp is responsible for formation of the MAM. The vWbp emp double mutant was complemented with plasmids encoding emp (pEmp) or vWbp (pvWbp) alone or both (pvWbpEmp) under their native promoters. This in trans approach led to overproduction of the respective proteins (compare Fig. S1). Expression of Emp from the plasmid caused increased size and more irregular shape of microcolonies but did not complement the lacking MAM. In contrast to this, expression of vWbp from the plasmid restored the MAM phenotype (blue arrowhead) and led to an increased diameter, possibly due to overproduction. Scale bar 150 µm. The images (A–C) are representative of three independent experiments. Data (D) are averaged from two independent experiments. (TIF) [file ppat.1002434.s002.tif]

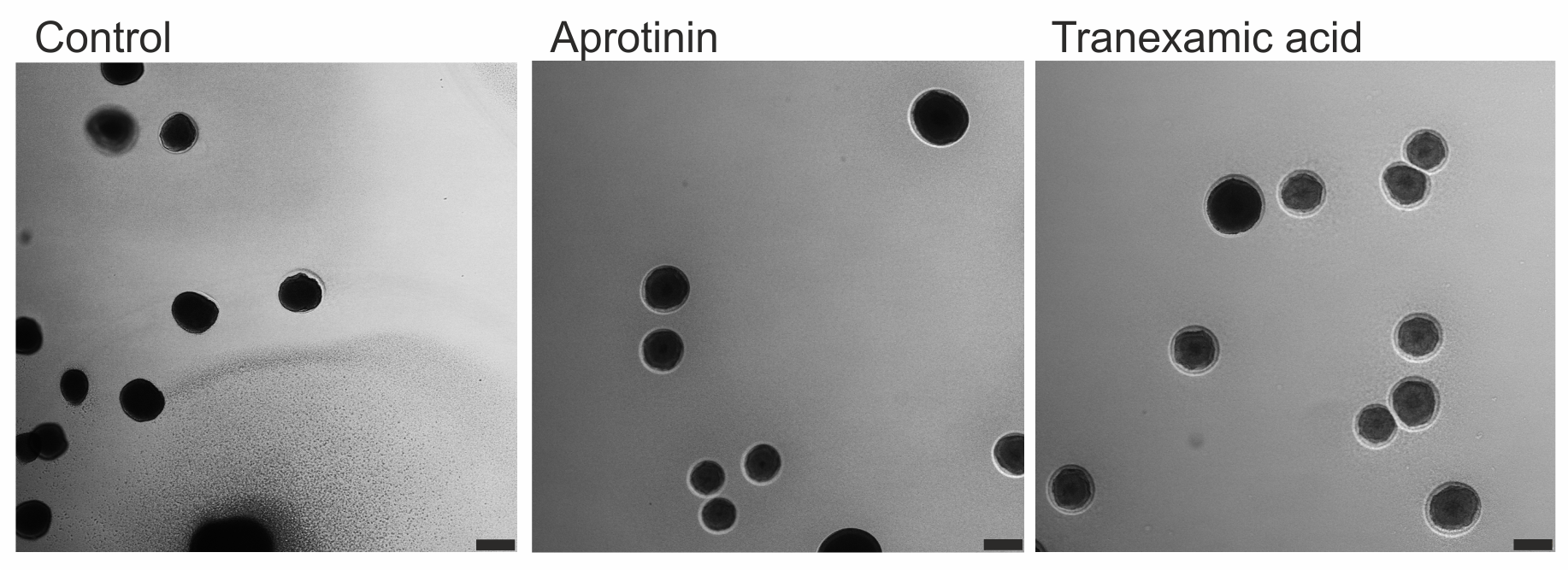

Supplement: Figure S3 — Inhibition of fibrin degradation by plasmin inhibitors. Aprotinin (final concentration 12,6 µM) or tranexamic acid (final concentration 3 mM) were added to the growth medium at t = 0h. This was repeated after 24h and 48h in order to compensate for possible decay of the inhibitor activity. Even after 6 days no fibrin degradation surrounding microcolonies could be observed. Three representative sections are shown. Scale bar 100 µm. (TIF) [file ppat.1002434.s003.tif]

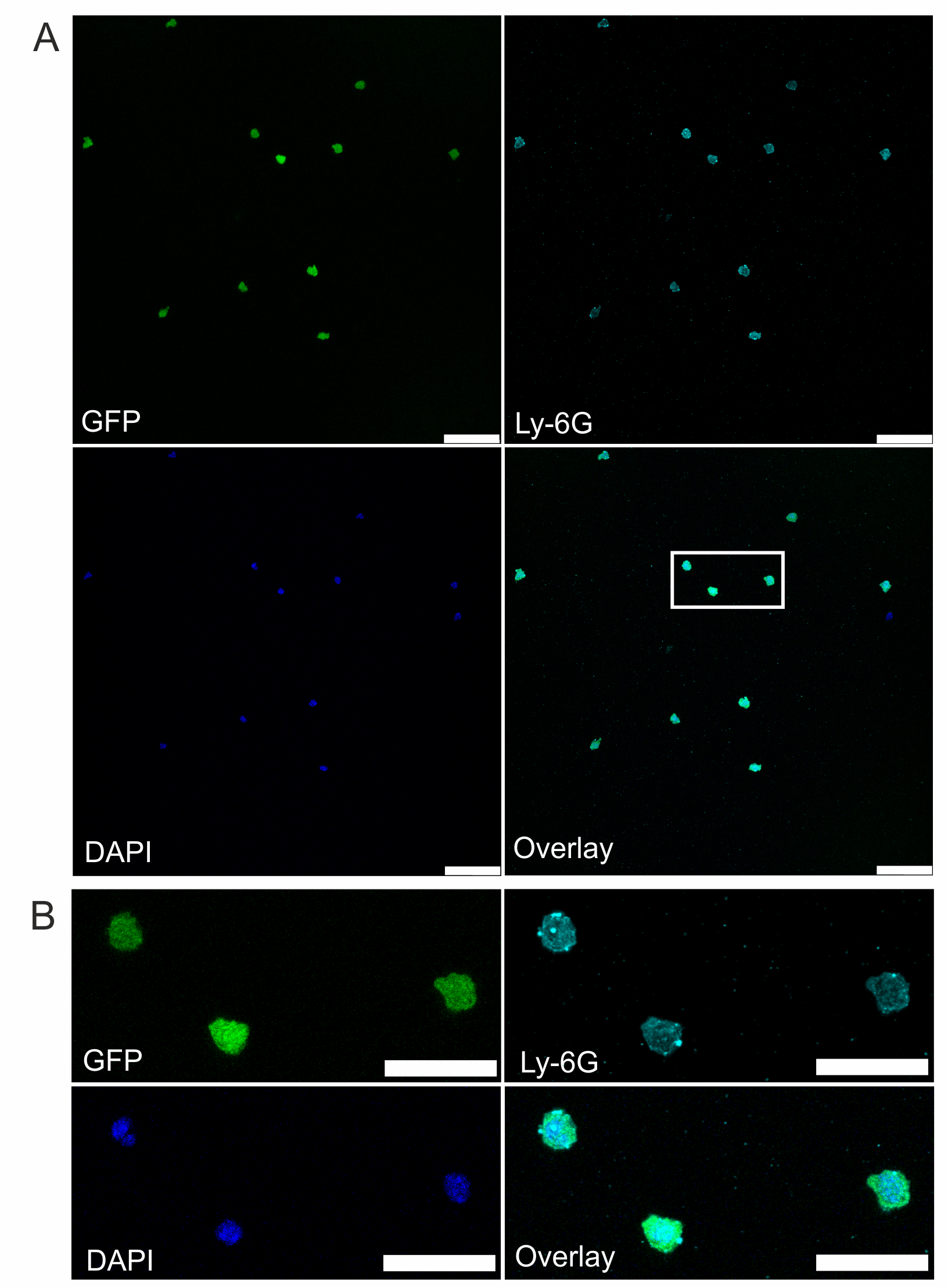

Supplement: Figure S4 — GFP+ cells migrating into 3D-CoG are Ly-6G+. Spleen slices from heterozygous lys-EGFP C57BL/6 mice were layered on top of preformed 30 µl 3D-CoG and incubated at 37°C for 4 h. Subsequently, the spleen slice was removed and cells inside the 3D-CoG were immunostained for Ly-6G. All GFP+ cells were Ly-6G+. An area including a GFP- cell is selected to include a negative Ly-6G- control cell. B is a magnification of the inset in A. A: scale bar 50 µm. B: scale bar 25 µm. (TIF) [file ppat.1002434.s004.tif]

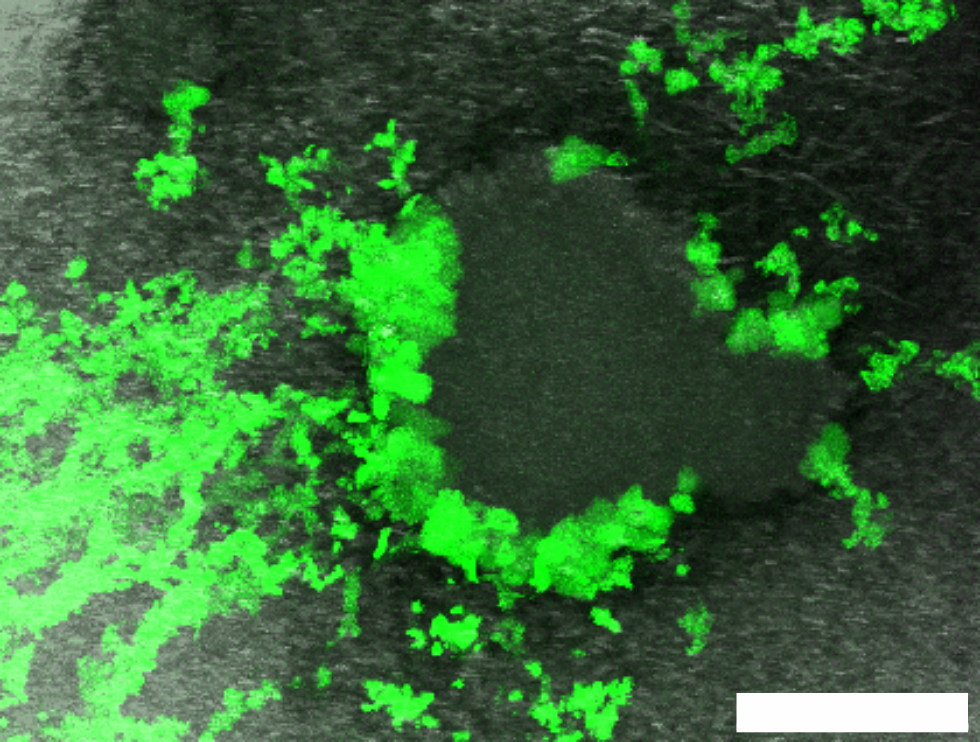

Supplement: Figure S5 — Time projection of interaction of neutrophils with Newman sae mutant in 3D-CoG/Fib. S. aureus sae mutant clusters grown in 3D-CoG/Fib for 17 h were invaded and phagocytosed by neutrophils without delay. Figure S5 shows a time projection of the entire observation period (87 min) of Video S4 (projection of two sections spanning a total depth of 5.3 µm). Green: GFP-neutrophils; White: confocal reflection microscopy showing collagen fibers. Scale bar 75 µm. (TIF) [file ppat.1002434.s005.tif]
