# Supplementary material for: Two Distinct Coagulase-Dependent Barriers Protect Staphylococcus aureus from Neutrophils in a Three Dimensional in vitro Infection Model
Source: PLoS Pathog. 2012 Jan 12;8(1):e1002434. doi: 10.1371/journal.ppat.1002434 (PMC3257306; doi:10.1371/journal.ppat.1002434)
Supplement: Table S1 — Oligonucleotides used in this study. (DOC) [file ppat.1002434.s006.doc]

Table S1: Oligonucleotides used in this study.

| Oligonucleotide | Sequence |
| --- | --- |
| vWbp-f-EcoRI | AAA GAATTC TAATGATATTAAATTAATCATATG |
| vWbp-r-SalI | AAA GTCGAC AATTATTGATATTGATAGTTAAGC |
| vwb-r-BamHI | AAA GGATCC AATTATTGATATTGATAGTTAAGC |
| emp-f-BamHI | AAA GGATCC AAGCTGAAAAACAATAAAAATGTT |
| emp-r-PstI | AAA CTGCAG TGTTACTTCCAACTTTCAAAGTAG |
